# Cotton Late Embryogenesis Abundant (*LEA2)* Genes Promote Root Growth and Confer Drought Stress Tolerance in Transgenic *Arabidopsis thaliana*

**DOI:** 10.1534/g3.118.200423

**Published:** 2018-06-22

**Authors:** Richard Odongo Magwanga, Pu Lu, Joy Nyangasi Kirungu, Qi Dong, Yangguang Hu, Zhongli Zhou, Xiaoyan Cai, Xingxing Wang, Yuqing Hou, Kunbo Wang, Fang Liu

**Affiliations:** * State Key Laboratory of Cotton Biology/Institute of Cotton Research, Chinese Academy of Agricultural Sciences, Anyang 455000, China; † School of Biological and Physical Sciences (SBPS), Main Campus, Jaramogi Oginga Odinga University of Science and Technology (JOOUST), Main Campus, P.O. Box 210-40601 Bondo, Kenya

**Keywords:** LEA2 proteins, miRNAs, Drought stress, Expression analysis, Transgenic plant, Oxidants, Antioxidants

## Abstract

Late embryogenesis abundant (LEA) proteins play key roles in plant drought tolerance. In this study, 157, 85 and 89 candidate LEA2 proteins were identified in *G. hirsutum*, *G. arboreum* and *G. raimondii* respectively. *LEA2* genes were classified into 6 groups, designated as group 1 to 6. Phylogenetic tree analysis revealed orthologous gene pairs within the cotton genome. The cotton specific LEA2 motifs identified were E, R and D in addition to Y, K and S motifs. The genes were distributed on all chromosomes. LEA2s were found to be highly enriched in non-polar, aliphatic amino acid residues, with leucine being the highest, 9.1% in proportion. The miRNA, ghr-miR827a/b/c/d and ghr-miR164 targeted many genes are known to be drought stress responsive. Various stress-responsive regulatory elements, ABA-responsive element (ABRE), Drought-responsive Element (DRE/CRT), MYBS and low-temperature-responsive element (LTRE) were detected. Most genes were highly expressed in leaves and roots, being the primary organs greatly affected by water deficit. The expression levels were much higher in *G. tomentosum* as opposed to *G. hirsutum*. The tolerant genotype had higher capacity to induce more of *LEA2* genes. Over expression of the transformed gene *Cot_AD24498* showed that the *LEA2* genes are involved in promoting root growth and in turn confers drought stress tolerance. We therefore infer that *Cot_AD24498*, *CotAD_20020*, *CotAD_21924* and *CotAD_59405* could be the candidate genes with profound functions under drought stress in upland cotton among the *LEA2* genes. The transformed *Arabidopsis* plants showed higher tolerance levels to drought stress compared to the wild types. There was significant increase in antioxidants, catalase (CAT), peroxidase (POD) and superoxide dismutase (SOD) accumulation, increased root length and significant reduction in oxidants, Hydrogen peroxide (H_2_O_2_) and malondialdehyde (MDA) concentrations in the leaves of transformed lines under drought stress condition. This study provides comprehensive analysis of LEA2 proteins in cotton thus forms primary foundation for breeders to utilize these genes in developing drought tolerant genotypes.

Drought stress is one of the major abiotic stress factors with deleterious effects in plant growth and development (Sofia *et al.* 2013). With the ever changing environmental condition and erratic precipitation levels, plant production is projected to undergo further decline, that meeting the demands and needs of the growing population will be a challenge in the near future (Tilman *et al.* 2011). Plants being sessile, the effects caused by the various abiotic stresses are enormous thus threatening their existence (Rejeb *et al.* 2014). Plants have developed various coping strategies for continued survival under these extreme conditions, one of which is through the induction of various transcriptome factors (TFs) with the aim of boosting their tolerance level (Xiong and Ishitani 2006). One of the transcriptome factor (TF) that has a functional role under various abiotic stress conditions is a member of the late embryogenesis abundant (LEA) proteins (Rodriguez-Salazar *et al.* 2017). LEA proteins are basically grouped into eight (8) sub families, named as LEA1, LEA2, LEA3, LEA4, LEA5, LEA6, seed maturation proteins (SMPs) and dehydrins (Battaglia and Covarrubias 2013). In several studies conducted on the genome wide identification, the proteins encoding the late embryogenesis abundant (*LEA*) genes have been found to be the most abundant among all the other LEA protein families (Yang and Xia 2011).

LEA2 proteins are the members of a larger protein family of the late embryogenesis abundant (LEA) (Hundertmark and Hincha 2008). As the name suggests, this group of proteins are found to in large quantities in seeds at the late stages of embryo development (Dure *et al.* 1983). Even though, the LEA proteins are synonymous with the seeds, a number of LEA proteins have been detected in the other plant tissues, such as the vegetative tissues (de Nazaré Monteiro Costa *et al.* 2011). The distribution of LEA proteins is not restricted to plants only, but have been found in animals (10) (Denekamp *et al.* 2010) and in bacteria (11) (Espelund *et al.* 1992). The LEA protein families basically have universal structural architecture, high hydrophilicity, low proportion of cysteine (Cys) and tryptophan (Trp) residues and high contents of arginine (Arg), lysine (Lys), glutamate (Glu), alanine (Ala), threonine (Thr) and glycine (Gly). Due to the unique and common features of the LEA proteins, the LEA proteins are mainly referred as hydrophilins with a hydrophilicity index of more than 1 and a glycine (Gly) content of more than 6% (Battaglia *et al.* 2008).

The late embryogenesis abundant (LEA) proteins have been positively correlated with several of abiotic stress, and have been found to confer tolerance in plants such as *Brassica napus* (Dalal *et al.* 2009), rice (He *et al.* 2012) and *Fagus sylvatica* (Jiménez *et al.* 2008). For instance, overexpression of *Arabidopsis LEA* gene, *AtLEA3* have been found to enhance tolerance to drought and salinity stresses (Zhao *et al.* 2011). Overexpression of a rice *LEA* gene type, *OsLEA3-1* was found to confer drought tolerance (Xiao *et al.* 2007). Similarly, the *LEA* gene *HVA1 LEA* gene from barley, was found to confer dehydration tolerance in transgenic rice (Babu *et al.* 2004). In addition, *SiLEA14*, a novel gene was found to be highly expressed in the roots of foxtail millet under drought condition (Wang *et al.* 2014). However, the precise roles of LEA proteins are still not well understood. A number of proposals have been made to explain the possible roles of the LEA proteins in plants during water deficit conditions, such as enzyme protection (Hand *et al.* 2011), molecular shield (Furuki *et al.* 2011), hydration buffer (Hundertmark *et al.* 2012) and membrane interactions (Olvera-Carrillo *et al.* 2011). To date, a number of studies have been conducted in trying to determine the distribution and characterization of the LEA proteins in various plants, for instance *Arabidopsis* (Hundertmark and Hincha 2008), *Brassica napus* (Dalal *et al.* 2009), water melon (Celik Altunoglu *et al.* 2017) among other plants. Despite all the significance of the *LEA* genes, little has been done to investigate their putative role in cotton in relation to drought stress tolerance.

Cotton (*Gossypium hirsutum*) is an economically important fiber and oil crop cultivated in many tropical and subtropical areas of the world, where they are constantly exposed to a range of abiotic stresses which includes drought, extreme temperature and high salinity (Mahajan *et al.* 2005). The completion and publication of the draft genome sequences of upland cotton *G. hirsutum* (Li *et al.* 2015b), *Gossypium arboreum* (Li *et al.* 2015c) and *Gossypium raimondii* (Wang *et al.* 2012) has become a valuable tool in elucidating the transcriptome factors (TFs) in cotton genomes. There is a paucity of information available about LEA2 sub family in upland cotton. Therefore, in this study we carried out the identification, characterization of the *LEA2* genes in three cotton genomes and transformed a novel *LEA2* gene, *Cot_AD24498* into *Arabidopsis thaliana*, in which we further investigated the expression levels of the transformed gene in both the transgenic lines and the wild type (WT) under drought stress condition.

## Materials and methods

### Identification, Sequence Analysis, Phylogenetic Tree Analysis and Subcellular Location Prediction of The LEA2 Proteins In Cotton

*G. hirsutum*, tetraploid (AD) genome LEA2 protein sequences were downloaded from the Cotton Research Institute website (http://mascotton.njau.edu.cn). The *G. arboreum* of A genome LEA2 protein sequences were downloaded from the Beijing Genome Institute database (https://www.bgi.com/), and *G. raimondii* of D genome was obtained from Phytozome (http://www.phytozome.net/). The conserved domain of LEA2 protein (PF03168) was downloaded from Pfam protein families (http://pfam.xfam.org). The hidden Markov model analysis (HMM) profile of LEA2 protein was queried to carry out the HMMER search (http://hmmer.janelia.org/) (Finn *et al.* 2011) against *G. hirsutum*, *G. raimondii* and *G. arboreum* protein sequences. The amino acids sequences were analyzed for the presence of the LEA2 protein domains by ScanProsite tool (http://prosite.expasy.org/scanprosite/) and SMART program (http://smart.embl-heidelberg.de/). The three cotton genomes LEA2 proteins together with the LEA2 proteins from *Arabidopsis* (http://www.arabidopsis.org/) and rice (http://rice.plantbiology.msu.edu/index.shtml) were used to investigate the evolutionary history and patterning in relation to orthology or paralogy among the proteins encoding *LEA2* genes. A phylogenetic tree was constructed, the multiple sequence alignments of all the LEA2 proteins were done by Clustal omega, MEGA 7.0 software using default parameters as described by Higgins *et al.*, (Higgins *et al.* 1996). The physiochemical characteristics of all the obtained LEA2 proteins were determined through an online ExPASy Server tool (http://www.web.xpasy.org/compute_pi/). In addition, subcellular location prediction for all the upland cotton LEA2 proteins were determined through Wolfpsort (https://www.wolfpsort.hgc.jp/) (Horton *et al.* 2007). The subcellular prediction results were further validated through other two online tools TargetP1.1 server (Emanuelsson *et al.* 2007) and Protein Prowler Subcellular Localization Predictor version 1.2 (http://www.bioinf.scmb.uq.edu.au/pprowler_webapp_1-2/) (Bodén and Hawkins 2005).

### Analysis of promoter regions, chromosomal locations and miRNA target prediction of *LEA2* genes

To identify the presence of drought stress-responsive *cis*-acting regulatory elements in LEA2 promoter regions, 1 kb up and down stream region from the translation start site of the *LEA2* genes were analyzed using the PLACE database (http://www.dna.affrc.go.jp/place/signalscan.html) (Higo *et al.* 1999). The physical locations in base pair (bp) of each *LEA2* genes were determined through BLASTN searching against the local database. Mapchart software (https://www.wur.nl/en/show/Mapchart.htm) (Voorrips 2002), was used to plot the gene loci on *G. hirsutum*, *G. arboreum* and *G.raimondii* chromosomes. Finally we analyzed the miRNA targeting the *LEA2* genes by submitting all the coding sequences (CDS) of all the *LEA2* genes to the psRNATarget database (http://plantgrn.noble.org/psRNATarget/).

### Expression analysis of *LEA2* genes and determination of the gene to be transformed

The qRT-PCR analysis was used to determine the expression changes of the *LEA2* genes in response to drought stress in the two parental lines used. the upland elite cultivar, *G. hirsutum* is known to be drought sensitive while the wild tetraploid cotton, *G. tomentosum* is a drought tolerant (Zheng *et al.* 2016). The two cotton genotypes were treated for drought stress for 14 days. The samples for RNA extraction were obtained from the leaves, stem and roots, at 0, 7 and 14 days of stress exposure. All the samples were taken in three biological replicates in both control and treated seedlings. In order to get the best sets of the *LEA2* genes for carrying out qRT-PCR validation, we had to rely on the RNA-sequencing data profiled under drought stress condition. The RNA-Sequence data were downloaded from cotton research institute website (http://mascotton.njau.edu.cn/html/Data). RNAs were reversely transcribed to first strand cDNA by use of TransCript-All-in-One-First-Strand cDNA synthesis Super Mix for qPCR (TransGen, Beijing, China). The fluorescent quantitative primers were designed for the selected genes (24 up and 24 down regulated genes) using Primer Premier 5 (Supplemental Table S1). Actin gene served as a reference. The synthesized cDNA was pre-incubated at 95° for 15 sec, followed by 40 cycles of denaturation at 95° for 5 sec and extension at 60° for 34 sec. The fluorescence quantitative assay was used to analyze expression level of the *LEA2* genes in root, leaves and stem tissues of cotton plant, and expression changes in *G. hirsutum* and *G. tomentosum* under drought stress. The assay was designed with three replicates and the results were analyzed with the double delta Ct method.

### Transformation and Screening of Novel gene *Cot_AD24498* (LEA2) in the Model Plant *Arabidopsis thaliana* (Ecotype Colombia-0) Lines

The gene was transformed into model plant, *A. thaliana* ecotype Colombia-0 (Col-0). The upland cotton, *G. hirsutum*, accession number CRI-12 (G09091801–2) was used to confirm for the presence of the *Cot_AD24498* gene in various tissues. The pWM101-35S:*Cot_AD24498* (LEA2) construct in *Agrobacterium tumefaciens* GV3101 was confirmed by gene specific primer, the forward primer sequence *Cot_AD24498* (5′CGGATCCATGTCGGTAAAAGAGTGCGGC3′) and reverse primer sequence pair of *Cot_AD24498* (5′GGTCGACTTACACGCTAACACTGCATCT3′), synthesized from Invitrogen, Beijing, China. The *Arabidopsis* Wild-type (WT) plants were transformed by use of floral dip method (Clough SJ und Bent A 1998). Infiltration media mainly composed of 4.3 g/l, sucrose 50 g/l (5%), 2-(4-morpholino) ethane sulfonic acid (MES) 0.5 g/l, Silwet-77 200 µl/l (0.02%), 6-benzylaminopurine (6-BA) 0.01 mg/l with pH of 5.7. Transformed lines of *A. thaliana* were selected by germinating seeds on 50% (0.5) MS (PhytoTechnology Laboratories, Lenexa, USA), containing 50 mg/l hygromycin B (Roche Diagnostics GmbH, Mannheim, Germany) for a duration of three (3) days at temperature of 4° to optimize germination. Upon which the seedlings were transferred to *Arabidopsis* conditioned growth room set at 16 hr light and 8 hr dark. After 7 days in selection medium, and at three true leaves stage, the seedlings were transplanted into small plastic containers filled with vermiculite and humus in equal ratios. The seedlings at generation T0 were grown to set seeds, the seeds obtained were generation T1. The T1 seeds were germinated in selective antibiotic medium; the one-copy lines were identified by determining the segregation ratio of 3:1 of the antibiotics-selectable marker. The 3:1 ratio of the segregated lines (T2) seeds were again germinated in antibiotics-selective medium, only the lines with 100% were selected for the development of T3 generation. The T3 homozygous progeny was bred from a T2 population after real-time quantitative reverse transcription polymerase chain reaction (qRT-PCR) and the selection of three out of the eight successfully transformed overexpressed lines (L2, L3, and L4) was done by using *Cot_AD24498 (LEA2)* forward primer sequence (5′CGAACATCCATCCCTCCAAC3′) and *Cot_AD24498 (LEA2)* reverse primer sequence (5′ATCATCAAGAAAACCGACCC3′) with total complementary DNA (cDNA) as template. The phenotypic investigations were carried out in T3 homozygous generation.

### qRT-PCR Analysis of the Expression of Drought-Responsive Genes in Transgenic *Arabidopsis*

We assessed the action of the transformed gene in the transgenic lines and the wild type of the model plant, *A. thaliana* by carrying out expression analysis of two drought responsive genes. ABRE-binding factor 4 (*ABF4)* gene; forward sequence 5′AACAACTTAGGAGGTGGTGGTCAT3′ and reverse sequence 5′TGTAGCAGCTGGCGCAGAAGTCAT3′ and responsive to desiccation 29A *(RD29A)* gene with forward sequence 5′TGAAAGGAGGAGGAGGAATGGTTGG3′ and the reverse sequence 5′ACAAAACACACATAAACATCCAAAGT3′. Total RNA was isolated from four-week-old transgenic *Arabidopsis* seedlings and wild type (Columbia ecotype) grown under normal conditions (CK) and 15% PEG6000 treatments for 4 days. RNA extraction and real-time RT-PCR (qRT-PCR) analyzed was applied as described in the section” Expression analysis of *LEA2* genes and determination of the gene to be transformed”, cotton *Actin2* forward sequence 5′ATCCTCCGTCTTGACCTTG3′ and reverse sequence 5′TGTCCGTCAGGCAACTCAT3′ applied as the reference gene.

### Quantification of oxidant and antioxidants in transgenic lines and the wild type

When plants are exposed to any form of stress, there are drastic changes which occurs both at molecular and cellular level in order to tolerate the stress factors (Gill *et al.* 2016). Reactive oxygen species is an oxidant substance being produced continuously from the respiring cells, and plants have an elaborate mechanism to keep the level within nontoxic limit, but when stresses such as drought sets in, the ROS equilibrium shifts leading to excessive production. In this research work, we undertook to evaluate the various oxidants and antioxidants levels between the transgenic lines (L1, L2 and L3) compared to the wild type when exposed to drought stress condition. Catalase (CAT), superoxide dismutase (SOD), peroxidase (POD), Malondialdehyde (MDA) and hydrogen peroxide (H_2_O_2_) levels were quantified according to the method described by Bartosz (Bartosz 2005). The seeds for transgenic and the wild types were grown in0.5 MS for eight (8) days, then transferred to small conical containers filled with a mixture vermiculite and sand in the ratio of 1:1 and grown for 21 days. After 21 days, water was totally withdrawn from drought treated plants for a period of 8 days, while the controlled plants were watered normally. The leaf samples were then harvested for antioxidants and oxidant determination after 8 days of post stress exposure. The samples were obtained in triplicate, in which each represented a biological repeat.

### Availability of Data Statement

The author do affirms that all the data supporting the conclusions of this research work are represented fully within the manuscripts and its supplementary files. Supplemental material available at Figshare: https://doi.org/10.25387/g3.6626849.

## Results and discussion

### LEA2 protein encoding genes in the cotton genome and other plants

In the identification of the LEA2 proteins in the three cotton genomes, we employed the Hidden Markov Model (HMM profile) of the Pfam LEA2 domains PF03168, as keyword to search the three cotton genome sequences databases. Based on the Pfam domain search, we obtained 200 *LEA2* genes in *G. hirsutum* of AD genome, 101 *LEA2* genes in *G. raimondii* of D genome and 110 *LEA* genes in *G. arboreum* of A genome. In order to ascertain the various genes obtained for the three cotton genomes, we carried out manual search through SMART (http://smart.embl.de/smart/) and PFAM database (http://pfam.xfam.org) to verify the presence of the *LEA2* gene domain. Upon removal of the redundant sequences with no functional domain or those that lacked the LEA2 domains, we eventually obtained 157, 85 and 89 LEA2 proteins in *G. hirsutum*, *G. arboreum* and *G. raimondii*, respectively. The confirmed domains of the LEA2 proteins in the three cotton genomes were further analyzed for their functional domain attributes of the LEA2 proteins, by use of an online tool, conserved domain database (CDD) tool hosted in the NCBI database. The results showed that the LEA2 proteins were members of *c112118* super family with E values ranging from 0 to 0.008 (Supplementary Table S2) and all contained transmembrane domain (Supplementary Table S3) The association of the LEA2s with transmembrane domain could possibly explain the reason why the LEA proteins are found in high concentrations in seeds at late stages of seed development, this possibly to aid in maintaining the stability of the cell membrane under dehydration state. Similar results have also been reported in some of the drought and salt enhancing genes such as Salicornia brachiata SNARE-like superfamily protein (*SbSLSP*), has been reported to be localized in the plasma membrane (Singh *et al.* 2016). LEA2 proteins could be playing an integral role in maintaining non-lethal level of reactive oxygen species (ROS homeostasis) in order to minimize oxidative damages to cellular membranous and macromolecules, in addition, LEA2s could also be playing similar roles as the aquaporin’s, the water channel proteins, which are responsible in the regulation of water movement channels such as plasmodesmata and xylem vessels (Buckley 2015). Aquaporin’s (AQPs) have been associated with salt and drought stress tolerance in plants, the aquaporin’s share similar functional domain with LEAs, being basically membrane proteins (Li *et al.* 2015a).

The number of proteins encoding the *LEA2* genes found in *G. arboreum*, *G. raimondii* and *G. hirsutum* were relatively higher than the number recorded in other plants, the entire repertoire of LEA proteins in the 8 LEA families outlined in (Hundertmark and Hincha 2008) have been found to be 34 in rice (Wang *et al.* 2007), 30 in Chinese plum (Du *et al.* 2013), 27 in tomatoes (Cao and Li 2014), 53 in poplar (Lan *et al.* 2013) and 29 in potatoes (Charfeddine *et al.* 2015), which is far below the individual numbers of LEA2 in the three cotton genome. The abundance of cotton proteins encoding the *LEA2* genes could be possibly due to their unique characteristics of being more hydrophobic than other LEA2 proteins from other species and or they could have evolved much later after other transcriptome factors. The genome size of plants and animal is constant, and high abundance of a particular gene family gives an indication of their integral role in enhancing the survival of the plants. The ever changing environmental conditions, plants are constantly faced with hearse environmental condition and disadvantaged by their sessile nature. The survival of the plants under these extreme environmental conditions therefore is through the increase of more stress tolerance genes or integrating a more complex gene interaction in initiating adaptive response mechanisms aimed at increased tolerance levels (Avramova 2015).

### Phylogenetic analyses of LEA2 proteins in *G. hirsutum*, *G. arboreum and G. raimondii*

Phylogenetic tree analysis provides valuable knowledge on the lines of evolutionary descent of different genes or proteins from a common ancestor, since its inception, it has remained a powerful tool for structuring classifications, biological diversity and for providing insight into events that occurred during gene evolution (Gregory 2008). In this study a total of 157, 85 and 89 LEA2 proteins were identified from *G. hirsutum*, *G. arboreum* and *G. raimondii*, respectively (Table 1). All the LEA2 proteins were aligned by the neighbor joining (NJ) method in ClustalW. The various LEA2 proteins from upland cotton, G. arboreum, G. raimo*ndii*, *A. thaliana*, *T. cacao and G. max* were analyzed. The inclusion of *A. thaliana*, *T. cacao and G. max* in the analysis of the cotton LEA2s was due to fact that *Theobroma cacao* share ancestral origins with cotton, *A. thaliana* and *G. max* have undergone whole genome duplication similar to cotton plant. The resulting phylogenetic tree showed that the cotton *LEA* genes tend to cluster together. Based on the clustering pattern, the *LEA2* genes were sub-divided into 6 groups, namely group 1 with three sub-groups, group 2, group 3 with two sub-groups, group 4, group 5 and finally group 6 with 5 sub-groups. Groups 1, 2, 4 and 5 were entirely LEA2 proteins from the three cotton genomes.

**Table 1:** The identified *LEA2* genes and their nomenclatural description

In this work	Hundertmark & Hincha (2008)	*G. hirsutum*	*G. arboreum*	*G. raimondii*	*V. vinifera*	*B.napus*	*G. max*	*Arabidopsis*
LEA2	LEA_2	157	85	89	1	4	5	3

The LEA2s seems to have evolved later among all the *LEA* genes, in the analysis of the *LEA* genes in sweet orange, the highest among all the 8 members of the *LEA* genes were members of the LEA2 (Muniz Pedrosa *et al.* 2015), this kind of observation was replicated in a number of plants. More than a half of the phylogenetic tree was mainly covered by the cotton LEA2 proteins, with no presence of LEA2s from other plants used in the analysis of the phylogenetic tree. *Theobroma cacao*, being evolutionary related to cotton, a few members of the LEA proteins clustered with cotton, while majority of the proteins encoding the *LEA2* genes from *Theobroma cacao* clustered together.

**Figure 1:**
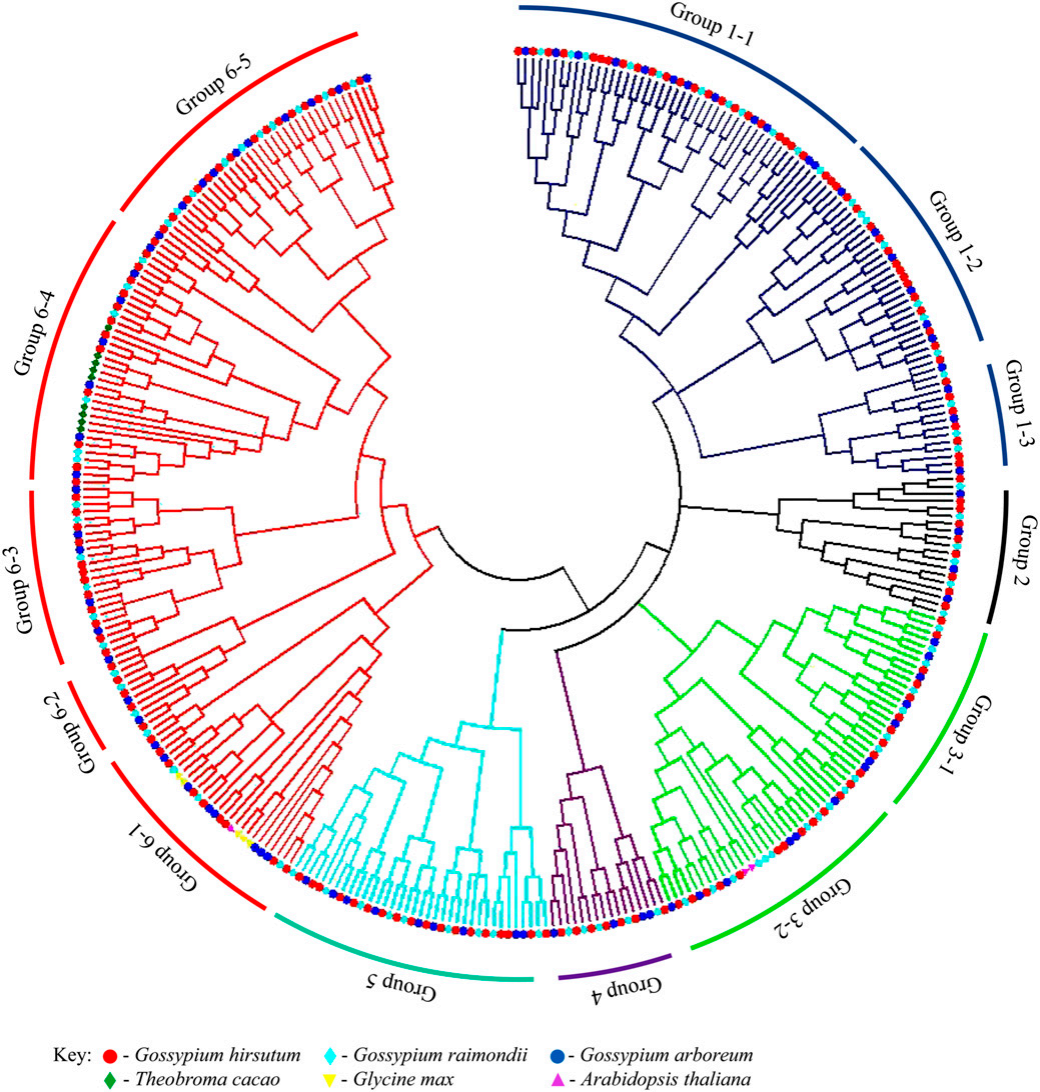
Phylogenetic relationship of *LEA2* genes in three cotton species with *Arabidopsis*, *T. cacao* and *G. max*. Neighbor-joining phylogeny of 157 genes for *G. hirsutum*, 85 genes for *G. arboreum*, 89 genes for *G. raimondii*, 9 genes for *T. cacao*, 5 *G. max* and 3 *Arabidopsis* LEA protein sequences, as constructed by MEGA7.0.

The late embryogenesis abundant (LEA2) proteins from *A. thaliana* were found to cluster with those of cotton LEA2s in group 3 and 6 (3-2 and 6-1) while *Glycine max* LEA2 proteins were predominantly found in group 6-1 (Figure 1). No ortholog gene pairs were detected between the proteins encoding the cotton *LEA2* genes of cotton to any of the plants used. All the ortholog gene pairs occurred between *G. hirsutum* and *G. arboreum*, *G. hirsutum* and *G. raimondii* and *G. arboreum* and *G. raimondii*. Interestingly, even *Theobroma cacao*, which is evolutionary related to *Gossypium* species, had their LEA2 proteins clustered together.

The abundance of LEA2s in plants can be explained by either being the last members of the *LEA* genes to evolve and or due to duplication. Upland cotton is a tetraploid cotton, having emerged through whole genome duplication (WGD) between the two diploid cotton of A and D genomes. The high number of *LEA2* genes, have also been observed in *Arabidopsis* (Hundertmark and Hincha 2008). Therefore, we could infer that LEA2 proteins might have evolved later after species divergence and the presence of ortholog genes in the cotton genome could be due to the whole genome duplication event coupled with chromosome rearrangement. It is generally assumed that ortholog genes have the same biological functions in different species (Tatusov 1997), and duplication makes room for paralogous gene pairs to evolve new functions (Ohno 1970). *LEA2* genes could be functionally-oriented ortholog groups consisting of orthologous pair which plays the same biological role in the three different cotton genomes.

### Physio-chemical analysis, subcellular localization and amino acid composition of the *LEA2* genes in upland cotton

In the analysis of the physio-chemical properties of the *LEA2* genes in upland cotton, the proteins encoding the *LEA2* genes had varied molecular formulae though with similar elemental composition, carbon (C), hydrogen (H), oxygen (O), nitrogen (N) and sulfur (S) in varying proportions. Molecular weights ranged from 11.5384 to 73.5831 kD, Pl values from 4.63 to 10.35, aliphatic index from 19.78 to 65.4, instability index from 6.91 to 63.52, protein lengths ranged from 100 to 661 bp and the grand average of hydropathy (GRAVY) values ranged from 0.574 to 1.04. The grand average hydropathy (GRAVY) values showed that almost all the LEA2s are hydrophobic proteins, the hydrophobic nature of proteins is integral for their biological functions, allows the proteins to fold spontaneously into complex three-dimensional structures that are significant for biological activity (Gosline *et al.* 2002). The hydrophobic nature of the proteins enables the removal of nonpolar amino acids from solvent and their burial in the core of the protein, this attribute is common among the aquaporin’s (AQPs), water channel proteins, are highly hydrophobic and known to have a functional role in water and salt stress tolerance in plants (Sreedharan *et al.* 2013). In the sub cellular localization prediction, 10 different sites were detected, in which majority of the LEA2 proteins were found to be localized within the chloroplast with 73 genes. Further analysis by TargetP and Pprowler, more than 70% of the genes were found to be associated with secretory pathway and chloroplast (Table 2 and Supplementary Table S4). The high number of these genes in chloroplast explains their significant role in drought stress, since chloroplast plays a central role in plant response to stress (Gläßer *et al.* 2014). The connection between different stress responses and organellar signaling pathways such as reactive oxygen species, emanate from the chloroplast (Kmiecik *et al.* 2016). Chloroplasts being semi-autonomous organelles provide complex communication channel that allow for effective coordination of gene expression since most plastid localized proteins are nuclear-encoded, thus ensuring an effective functioning of overall cellular metabolism (Pfannschmidt *et al.* 2009). Numerous and vital cellular processes such as aromatic amino acids, fatty acids and carotenoids biosynthesis and sulfate assimilation pathways are harbored within the chloroplast, in addition to photosynthesis, these cellular processes are known to be key factors in plants response to stress. The chloroplast acts as a sensor to abiotic stress thus initiates different cell functions in response to stress factor, enhancing adaptability of the plant to the environmental stress (Mittler 2006). Higher proportions of *LEA2* genes were found to be localized within the cytoplasm, nucleus and mitochondrion, with 24, 20 and 16 genes respectively, which further provided a stronger evidence of the importance of these genes in enhancing drought tolerance ability in cotton. The following cell structures contained low numbers of *LEA2* genes, endoplasmic reticulum (E.R) with 3, extracellular structures with 5, Golgi body 6, plasma 4 and vacuole with 3 genes each. The result obtained for the subcellular localization of the *LEA2* genes is in agreement to previous findings in which the highest proportions of *LEA2* genes were found to be localized within the cytoplasm and chloroplast, accounting for 35.7% and 30.9% of the total *LEA2* genes in sweet orange, while others were found to target endoplasmic reticulum (E.R) and mitochondrion (Muniz Pedrosa *et al.* 2015). Similarly, abiotic stress related gene, plasma membrane protein 3 (*PMP3*), a member of the small hydrophobic polypeptides with high sequence similarity, and have been functionally characterized to be responsible for salt, drought, cold, and abscisic acid, have been found to be sub localized in the nucleus, cytoplasm, and cell membrane (Fu *et al.* 2012).

**Table 2:** Physiochemical properties of *LEA2* gene in upland cotton, *G. hirsutum*, subcellular location prediction and chromosome position

Gene Id	Molecular Formula	Atoms Numbers	Instability Index	Aliphatic Index	Gravy	Length (Aa)	Pl	Mw (Aa)	Chr No	Sub Cellular Localization		
										Wolfpsort	TargetP	Prowler
**CotAD_ 00275**	C_2550_H_4266_N_832_O_1061_S_220_	8929	49.24	24.58	0.824	274	10	29834.66	Dt09_chr23	chlo	S	sp
**CotAD_ 00465**	C_2809_H_4694_N_922_O_1183_S_186_	9794	38.68	27.5	0.704	304	10	33689.28	Dt09_chr23	chlo	C	sp
**CotAD_ 00799**	C_3119_H_5215_N_1021_O_1297_S_196_	10848	42.14	31.89	0.776	337	9	38982.02	scaffold26.1	golg	C	sp
**CotAD_ 00808**	C_2114_H_3538_N_688_O_893_S_149_	7382	38.49	25.51	0.698	226	10	26011.22	scaffold26.1	cyto	_	sp
**CotAD_ 01033**	C_1868_H_3118_N_616_O_781_S_132_	6515	37.57	27.69	0.749	202	9	22587.14	Dt10_ch20	chlo	S	sp
**CotAD_ 01298**	C_1996_H_3326_N_664_O_833_S_142_	6961	35.29	27.79	0.754	218	10	24021.4	Dt10_ch20	cyto	_	other
**CotAD_ 01321**	C_2138_H_3550_N_724_O_880_S_189_	7481	48.5	25.62	0.855	238	10	26020.28	Dt10_ch20	cyto	S	sp
**CotAD_ 01385**	C_2253_H_3753_N_751_O_944_S_189_	7890	53.4	22.96	0.757	247	7	27497.03	Dt09_chr23	cyto	S	sp
**CotAD_ 01700**	C_2382_H_3972_N_790_O_976_S_223_	8343	52.12	25.76	0.914	260	9	28399.83	Dt09_chr23	cyto	_	sp
**CotAD_ 02652**	C_2022_H_3396_N_646_O_835_S_184_	7083	63.52	25.47	0.898	212	10	23764.43	Dt09_chr23	mito	S	sp
**CotAD_ 03037**	C_2465_H_4132_N_796_O_1011_S_239_	8643	54.69	25.19	0.943	262	9	28472.57	Dt05_chr19	cyto	S	sp
**CotAD_ 03649**	C_2938_H_4904_N_970_O_1220_S_232_	10264	42.02	27.07	0.811	320	10	35345.6	At_chr09	cyto	S	sp
**CotAD_ 03784**	C_1076_H_1792_N_358_O_453_S_53_	3732	26.71	31.74	0.644	116	7	13537.66	Dt07_chr16	chlo	_	other
**CotAD_ 05724**	C_1834_H_3065_N_601_O_771_S_128_	6399	46.85	26.71	0.719	197	10	22442.51	At_chr09	chlo	_	sp
**CotAD_ 05725**	C_2229_H_3732_N_724_O_935_S_169_	7789	50.4	25.76	0.755	238	10	27552.78	At_chr09	nucl	_	sp
**CotAD_ 06037**	C_1893_H_3159_N_625_O_802_S_134_	6613	45.22	24.24	0.668	205	10	22125.81	Dt13_ch18	chlo	_	sp
**CotAD_ 07087**	C_1926_H_3222_N_628_O_819_S_106_	6701	43.9	28.43	0.622	206	10	22853.64	At_chr02	plas	_	other
**CotAD_ 08181**	C_1864_H_3110_N_616_O_780_S_135_	6505	43.71	26.87	0.745	202	9	22460.02	Dt09_chr23	cyto	S	sp
**CotAD_ 08350**	C_1894_H_3182_N_604_O_790_S_142_	6612	49.06	27.91	0.802	198	5	22266.98	scaffold190.1	chlo	_	sp
**CotAD_ 08837**	C_2300_H_3853_N_745_O_961_S_220_	8079	55.29	20.73	0.825	245	9	26376.34	scaffold280.1	golg	S	sp
**CotAD_ 09578**	C_2381_H_3970_N_790_O_977_S_223_	8341	50.46	25.38	0.905	260	9	28406.84	At_chr09	chlo	_	sp
**CotAD_ 09685**	C_2306_H_3847_N_763_O_928_S_220_	8064	61.17	29.7	1.024	251	10	27153.8	Dt09_chr23	chlo	_	sp
**CotAD_ 09732**	C_2198_H_3688_N_706_O_923_S_164_	7679	47.07	26.14	0.755	232	9	25906.5	Dt09_chr23	chlo	C	sp
**CotAD_ 10376**	C_2568_H_4293_N_841_O_1038_S_271_	9011	60.05	25.86	1.033	277	10	30152.74	Dt01_chr15	chlo	S	sp
**CotAD_ 11658**	C_2438_H_4075_N_799_O_1007_S_165_	8484	34.86	31.99	0.823	263	10	29835.19	Dt08_chr24	cyto	_	sp
**CotAD_ 11875**	C_1627_H_2717_N_535_O_682_S_94_	5655	33.71	30.96	0.706	175	7	20070.28	scaffold42.1	chlo	S	sp
**CotAD_ 11876**	C_1942_H_3245_N_637_O_798_S_180_	6802	50.01	25.51	0.904	209	10	23563.32	scaffold42.1	chlo	_	other
**CotAD_ 11878**	C_2121_H_3552_N_688_O_886_S_165_	7412	55.97	26.24	0.785	226	10	25841.73	scaffold42.1	chlo	S	sp
**CotAD_ 11879**	C_1215_H_2031_N_397_O_519_S_61_	4223	41.32	28.35	0.574	129	10	15037.05	scaffold42.1	chlo	S	sp
**CotAD_ 12375**	C_1765_H_2948_N_580_O_727_S_157_	6177	61.3	25.95	0.879	190	9	21328.78	At_chr09	chlo	_	other
**CotAD_ 13115**	C_1791_H_2994_N_586_O_760_S_122_	6253	39.07	24.83	0.659	192	9	20770.35	Dt08_chr24	extr	_	sp
**CotAD_ 13584**	_2310_H_3858_N_760_O_957_S_190_	8075	46.59	26.65	0.832	250	10	28048.83	Dt06_chr25	golg	S	sp
**CotAD_ 13827**	C_3342_H_5592_N_1090_O_1370_S_299_	11693	55.07	27.48	0.922	360	8	40945.87	Dt12_ch26	E.R.	_	sp
**CotAD_ 14147**	C_2022_H_3396_N_646_O_838_S_180_	7082	61.99	25.16	0.871	212	10	23855.54	At_chr07	mito	S	sp
**CotAD_ 15892**	C_2861_H_4789_N_931_O_1209_S_186_	9976	40.47	27.23	0.688	307	8	34741.21	Dt12_ch26	chlo	_	sp
**CotAD_ 16731**	C_2370_H_3954_N_784_O_980_S_202_	8290	47.46	26.09	0.845	258	10	28519.44	Dt09_chr23	chlo	S	sp
**CotAD_ 17044**	C_1387_H_2309_N_463_O_581_S_100_	4840	43.02	26.25	0.725	151	5	16422.87	At_chr07	cyto	_	other
**CotAD_ 17045**	C_2199_H_3654_N_742_O_907_S_185_	7687	48.71	26.49	0.838	219	10	23930.18	At_chr07	cyto	_	other
**CotAD_ 17062**	C_2047_H_3416_N_676_O_852_S_170_	7161	50.56	25.07	0.802	244	10	27393.16	At_chr07	chlo	S	sp
**CotAD_ 17101**	C_1958_H_3277_N_637_O_811_S_177_	6860	53.57	24.41	0.86	222	9	25294.09	At_chr06	mito	_	sp
**CotAD_ 17102**	C_2435_H_4063_N_805_O_1008_S_182_	8493	41.98	29.02	0.817	209	10	23661.48	At_chr06	nucl	_	sp
**CotAD_ 17103**	C_2213_H_3709_N_715_O_930_S_187_	7754	61.46	22.72	0.767	265	7	30299.29	At_chr06	mito	_	sp
**CotAD_ 17649**	C_1849_H_3077_N_619_O_759_S_131_	6435	37.22	31.77	0.843	235	9	26726.9	At_chr10	chlo	S	sp
**CotAD_ 18210**	C_1850_H_3079_N_619_O_757_S_134_	6439	35.57	32.09	0.865	203	10	22501.33	scaffold377.1	cyto	_	other
**CotAD_ 18233**	C_1630_H_2729_N_529_O_675_S_118_	5681	40.59	29.98	0.822	203	10	22406.26	scaffold377.1	chlo	_	other
**CotAD_ 18546**	C_2571_H_4299_N_841_O_1038_S_270_	9019	58.81	26.34	1.04	173	10	19695.85	Dt09_chr23	chlo	_	sp
**CotAD_ 18729**	C_1990_H_3320_N_658_O_828_S_137_	6933	43.32	29.42	0.772	277	10	30227.97	scaffold336.1	chlo	S	sp
**CotAD_ 19078**	C_1684_H_2807_N_559_O_714_S_128_	5892	45.25	22.26	0.669	216	10	24007.7	At_chr12	nucl	S	sp
**CotAD_ 19107**	C_2766_H_4629_N_901_O_1165_S_184_	9645	42.16	27.59	0.71	183	9	20031.24	At_chr12	chlo	_	other
**CotAD_ 19205**	C_941_H_1570_N_310_O_394_S_56_	3271	35.65	30.19	0.703	297	7	33395.7	At_chr12	chlo	_	sp
**CotAD_ 19213**	C_1704_H_2853_N_553_O_707_S_109_	5926	45.8	32.12	0.793	100	10	11538.35	At_chr10	chlo	_	sp
**CotAD_ 19214**	C_2114_H_3541_N_685_O_887_S_125_	7352	35.76	30.89	0.719	181	9	20628.72	At_chr10	nucl	C	other
**CotAD_ 19375**	C_2310_H_3858_N_760_O_958_S_187_	8073	46.32	26.78	0.823	225	9	25956.2	Dt11_ch21	golg	S	sp
**CotAD_ 20020**	C_1807_H_3029_N_583_O_761_S_123_	6303	36.84	27.19	0.717	250	10	27947.68	At_chr06	mito	S	sp
**CotAD_ 20308**	C_2201_H_3658_N_742_O_909_S_184_	7694	46.29	26.35	0.83	191	10	21054.44	Dt06_chr25	chlo	_	sp
**CotAD_ 21731**	C_2426_H_4054_N_796_O_986_S_230_	8492	60.23	27.71	0.975	244	10	27381.21	Dt05_chr19	nucl	S	sp
**CotAD_ 21924**	C_1845_H_3069_N_619_O_756_S_138_	6427	38.31	30.96	0.86	262	10	28411.4	Dt11_ch21	nucl	S	sp
**CotAD_ 23646**	C_2458_H_4115_N_799_O_1036_S_200_	8608	53.5	22.84	0.738	204	10	21921.93	Dt07_chr16	nucl	_	other
**CotAD_ 24019**	C_1624_H_2711_N_535_O_680_S_94_	5644	33.71	31.14	0.711	203	10	22391.06	Dt06_chr25	mito	S	sp
**CotAD_ 24497**	C_1941_H_3243_N_637_O_796_S_181_	6798	50.1	25.83	0.916	263	9	29247.79	Dt10_ch20	chlo	S	sp
**CotAD_ 24499**	C_2118_H_3546_N_688_O_883_S_170_	7405	56.27	25.95	0.801	175	8	20026.25	scaffold238.1	chlo	_	sp
**CotAD_ 25271**	C_2240_H_3751_N_727_O_937_S_188_	7843	48.67	24	0.79	209	10	23559.33	scaffold238.1	nucl	S	sp
**CotAD_ 26038**	C_1695_H_2826_N_562_O_718_S_127_	5928	45.53	22.86	0.673	226	9	25852.71	scaffold238.1	chlo	_	sp
**CotAD_ 26981**	C_1423_H_2384_N_460_O_593_S_106_	4966	51.34	27.73	0.79	274	10	29936.66	At_chr09	chlo	C	sp
**CotAD_ 27453**	C_2034_H_3390_N_676_O_861_S_160_	7121	44.82	21.96	0.686	239	10	26994.13	scaffold477.1	mito	_	sp
**CotAD_ 27789**	C_2367_H_3951_N_781_O_998_S_201_	8298	53.3	21.31	0.731	184	9	20135.39	scaffold699.1	E.R.	_	sp
**CotAD_ 28249**	C_2260_H_3788_N_730_O_947_S_140_	7865	33.99	30.49	0.736	150	9	16764.6	At_chr09	nucl	_	sp
**CotAD_ 28252**	C_2177_H_3646_N_706_O_916_S_180_	7625	48.77	22.87	0.752	222	9	24982.77	At_chr07	mito	S	sp
**CotAD_ 28872**	C_1387_H_2306_N_466_O_578_S_109_	4846	48.91	25.43	0.764	257	9	26949.97	Dt03_chr17	nucl	_	sp
**CotAD_ 29279**	C_1875_H_3141_N_607_O_784_S_137_	6544	47.67	27.44	0.769	305	10	34588.47	Dt13_ch18	chlo	_	other
**CotAD_ 31344**	C_2277_H_3795_N_757_O_932_S_181_	7942	42.47	30.2	0.887	101	6	11711.01	scaffold1346.1	chlo	S	sp
**CotAD_ 31535**	C_2944_H_4916_N_970_O_1223_S_231_	10284	41.3	27.17	0.809	240	8	27649.86	At_chr05	vacu	S	sp
**CotAD_ 31536**	C_2047_H_3416_N_676_O_854_S_171_	7164	52.02	24.33	0.789	210	9	23875.63	scaffold1346.1	plas	S	sp
**CotAD_ 31537**	C_1956_H_3273_N_637_O_809_S_177_	6852	54.13	24.72	0.868	254	10	27558.52	scaffold1841.1	nucl	_	sp
**CotAD_ 31780**	C_2649_H_4422_N_874_O_1100_S_195_	9240	40.01	28.67	0.799	310	10	34525.38	Dt08_chr24	chlo	_	sp
**CotAD_ 31782**	C_1944_H_3258_N_628_O_812_S_139_	6781	46.14	28.27	0.774	210	8	23638.39	Dt09_chr23	chlo	S	sp
**CotAD_ 31860**	C_4139_H_6916_N_1360_O_1727_S_338_	14480	44.89	25.26	0.795	206	10	22839.69	scaffold257.1	cyto	_	sp
**CotAD_ 31906**	C_1914_H_3198_N_628_O_804_S_148_	6692	47.49	24.6	0.739	232	10	26256.38	scaffold769.1	cyto	C	sp
**CotAD_ 31936**	C_2627_H_4393_N_859_O_1089_S_219_	9187	47.93	26.37	0.838	152	5	16462.97	Dt01_chr15	mito	S	sp
**CotAD_ 32487**	C_1940_H_3238_N_640_O_815_S_167_	6800	51.19	21.79	0.753	305	10	33718.76	At_chr11	mito	_	sp
**CotAD_ 32645**	C_1845_H_3066_N_622_O_771_S_148_	6452	42.79	24.35	0.753	199	9	22785.41	Dt06_chr25	chlo	S	sp
**CotAD_ 32847**	C_1752_H_2928_N_574_O_730_S_100_	6084	39.49	32.87	0.745	249	10	27707.74	At_chr09	extr	S	sp
**CotAD_ 33143**	C_3449_H_5767_N_1129_O_1433_S_246_	12024	46.47	29.55	0.8	305	10	34544.43	Dt02_chr14	chlo	S	sp
**CotAD_ 33144**	C_1970_H_3298_N_640_O_818_S_163_	6889	54.12	26.18	0.83	240	9	27655.92	Dt05_chr19	chlo	_	sp
**CotAD_ 34476**	C_2374_H_3959_N_787_O_982_S_206_	8308	47.91	25.48	0.844	320	10	35579.84	Dt09_chr23	cyto	_	sp
**CotAD_ 34798**	C_2925_H_4884_N_964_O_1214_S_245_	10232	51.69	25.78	0.826	222	9	25253.03	Dt06_chr25	nucl	S	sp
**CotAD_ 35069**	C_2296_H_3827_N_763_O_944_S_159_	7989	48.49	32.19	0.84	209	10	23628.4	Dt06_chr25	chlo	S	sp
**CotAD_ 35091**	C_2037_H_3411_N_661_O_855_S_133_	7097	42.17	28.83	0.728	288	7	32755.52	Dt06_chr25	extr	_	sp
**CotAD_ 35514**	C_1704_H_2853_N_553_O_708_S_110_	5928	46.78	31.58	0.785	206	6	23420.27	Dt05_chr19	mito	S	sp
**CotAD_ 36328**	C_1970_H_3298_N_640_O_819_S_162_	6889	53.13	26.02	0.821	450	5	49131.5	scaffold821.1	chlo	C	other
**CotAD_ 36446**	C_1628_H_2725_N_529_O_673_S_119_	5674	44.25	30.17	0.833	231	10	24949.39	Dt08_chr24	chlo	_	other
**CotAD_ 36583**	C_2954_H_4936_N_970_O_1224_S_234_	10318	40.36	27.69	0.829	206	9	22761.2	scaffold821.1	chlo	_	sp
**CotAD_ 37776**	C_1843_H_3062_N_622_O_768_S_149_	6444	46.57	24.84	0.769	202	9	22357.93	Dt09_chr23	chlo	S	sp
**CotAD_ 37888**	C_2554_H_4274_N_832_O_1063_S_219_	8942	50.77	24.7	0.823	283	10	31410.18	At_chr08	chlo	S	sp
**CotAD_ 38978**	C_2819_H_4711_N_925_O_1184_S_205_	9844	42.1	26.22	0.734	210	10	22644.27	Dt08_chr24	nucl	S	sp
**CotAD_ 39064**	C_969_H_1623_N_313_O_399_S_81_	3385	56.09	27.65	0.874	210	10	23699.74	Dt01_chr15	chlo	S	sp
**CotAD_ 39719**	C_1971_H_3300_N_640_O_818_S_160_	6889	54.8	26.8	0.83	191	6	20961.07	Dt01_chr15	nucl	S	sp
**CotAD_ 40324**	C_2364_H_3954_N_772_O_959_S_228_	8277	54.49	27.79	0.995	204	10	21780.76	At_chr07	plas	_	sp
**CotAD_ 41569**	C_2875_H_4808_N_940_O_1188_S_244_	10055	50.55	26.76	0.862	208	10	22559.45	At_chr13	chlo	_	sp
**CotAD_ 41571**	C_1947_H_3252_N_640_O_803_S_171_	6813	59.76	26.02	0.87	270	10	30627.54	Dt09_chr23	chlo	_	sp
**CotAD_ 41925**	C_1928_H_3226_N_628_O_816_S_110_	6708	46.13	29.07	0.656	188	9	21941.4	scaffold1231.1	nucl	_	other
**CotAD_ 42599**	C_2794_H_4661_N_925_O_1169_S_206_	9755	43.38	26.65	0.752	373	10	43118.75	scaffold1231.1	cyto	_	other
**CotAD_ 44357**	C_2819_H_4711_N_925_O_1183_S_209_	9847	44.41	26	0.743	210	9	23874.6	scaffold1088.1	cyto	C	sp
**CotAD_ 45324**	C_2259_H_3786_N_730_O_944_S_141_	7860	34.69	31.04	0.754	256	10	28431.93	Dt11_ch21	chlo	S	sp
**CotAD_ 46873**	C_2117_H_3529_N_703_O_871_S_205_	7425	56.14	23.68	0.894	259	10	28603.52	At_chr09	vacu	S	sp
**CotAD_ 47322**	C_1862_H_3106_N_616_O_776_S_139_	6499	43.14	27.2	0.773	220	10	24666.72	At_chr03	chlo	S	sp
**CotAD_ 47454**	C_1973_H_3304_N_640_O_818_S_176_	6911	53.21	24.61	0.854	661	6	73583.12	scaffold1851.1	cysk	S	sp
**CotAD_ 47495**	C_1754_H_2923_N_583_O_719_S_178_	6157	55.01	23.06	0.922	318	10	35234.15	Dt07_chr16	chlo	S	sp
**CotAD_ 47749**	C_1922_H_3208_N_634_O_818_S_131_	6713	42.78	23.89	0.636	251	9	27769.63	Dt07_chr16	chlo	S	sp
**CotAD_ 48050**	C_2571_H_4320_N_820_O_1053_S_198_	8962	50.08	32.15	0.921	217	9	24968.87	Dt10_ch20	mito	_	sp
**CotAD_ 48069**	C_2356_H_3932_N_778_O_994_S_159_	8219	43.83	26.42	0.689	181	10	20577.73	Dt10_ch20	extr	S	sp
**CotAD_ 48336**	C_2036_H_3400_N_670_O_835_S_177_	7118	47.96	27.69	0.9	211	9	23479.93	Dt04_chr22	nucl	S	sp
**CotAD_ 48753**	C_6218_H_10441_N_1993_O_2614_S_448_	21714	47.81	27.02	0.752	210	9	23676.69	At_chr06	mito	_	sp
**CotAD_ 48769**	C_1998_H_3351_N_643_O_829_S_165_	6986	56.41	26.68	0.843	304	10	33675.21	At_chr09	nucl	_	sp
**CotAD_ 49818**	C_2811_H_4698_N_922_O_1186_S_183_	9800	36.96	27.39	0.691	317	5	35274.16	scaffold2616.1	cyto	S	sp
**CotAD_ 53045**	C_2922_H_4881_N_961_O_1224_S_173_	10161	37.17	30.97	0.72	206	8	22650.27	Dt10_ch20	cyto	S	sp
**CotAD_ 53263**	C_1938_H_3246_N_628_O_811_S_135_	6758	44.09	28.27	0.756	251	10	27168.81	At_chr09	chlo	_	other
**CotAD_ 53981**	C_2316_H_3867_N_763_O_933_S_219_	8098	61.7	29.83	1.021	247	7	27715.29	scaffold3326.1	mito	_	sp
**CotAD_ 54337**	C_2251_H_3749_N_751_O_943_S_189_	7883	54.62	22.96	0.757	152	5	16453.02	At_chr07	chlo	_	sp
**CotAD_ 55224**	C_1390_H_2312_N_466_O_579_S_109_	4856	50.61	25.65	0.768	210	10	23769.83	Dt03_chr17	mito	S	sp
**CotAD_ 56356**	C_1954_H_3266_N_640_O_822_S_101_	6783	33.97	32.13	0.677	173	10	19737.98	At_chr09	chlo	_	other
**CotAD_ 56696**	C_1963_H_3275_N_649_O_822_S_113_	6822	33.29	31.22	0.71	213	10	23750.48	Dt03_chr17	nucl	S	sp
**CotAD_ 58358**	C_1600_H_2547_N_445_O_483_S_11_	5086	61.19	65.4		209	10	23626.51	Dt12_ch26	chlo	S	sp
**CotAD_ 59405**	C_1936_H_3233_N_637_O_793_S_189_	6788	54.41	24.72	0.93	320	10	35457.72	Dt05_chr19	chlo	_	sp
**CotAD_ 60279**	C_2316_H3_879_N_751_O_968_S_220_	8134	53.86	20.83	0.82	247	9	26619.63	scaffold2414.1	chlo	S	sp
**CotAD_ 60435**	C_2292_H_3819_N_763_O_938_S_163_	7975	49.59	32.72	0.869	251	10	27952.81	At_chr01	chlo	S	sp
**CotAD_ 60617**	C_1977_H_3312_N_640_O_820_S_177_	6926	54.15	24.45	0.855	210	10	23780.9	Dt01_chr15	mito	S	sp
**CotAD_ 61173**	C_1964_H_3271_N_655_O_821_S_137_	6848	38.49	27.72	0.739	215	10	24043	At_chr04	chlo	_	other
**CotAD_ 61391**	C_1753_H_2921_N_583_O_718_S_179_	6154	54.1	23.06	0.928	191	6	20884.97	Dt01_chr15	chlo	S	sp
**CotAD_ 62996**	C_2926_H_4886_N_964_O_1214_S_245_	10235	51.58	25.88	0.828	318	10	35356.25	At_chr01	nucl	S	sp
**CotAD_ 63174**	C_3526_H_5909_N_1141_O_1460_S_281_	12317	44.13	28.18	0.85	377	10	41228.93	scaffold3177.1	E.R.	S	sp
**CotAD_ 64004**	C_2020_H_3371_N_667_O_833_S_157_	7048	48.11	29.02	0.845	219	10	23825.02	Dt07_chr16	chlo	_	sp
**CotAD_ 64120**	C_2001_H_3336_N_664_O_837_S_143_	6981	33.36	27.19	0.743	218	10	24050.43	At_chr12	chlo	S	other
**CotAD_ 64346**	C_1963_H_3284_N_640_O_817_S_168_	6872	52.92	24.61	0.818	210	9	23572.5	Dt06_chr25	chlo	_	other
**CotAD_ 64347**	C_2142_H_3567_N_715_O_883_S_231_	7538	59.98	19.78	0.901	235	9	26111.93	Dt06_chr25	plas	_	sp
**CotAD_ 64657**	C_2431_H_4064_N_796_O_990_S_225_	8506	59.04	27.96	0.961	262	10	28516.58	At_chr11	vacu	_	sp
**CotAD_ 65119**	C_1908_H_3186_N_628_O_800_S_147_	6669	42.93	25.08	0.747	206	9	22733.19	Dt08_chr24	golg	S	sp
**CotAD_ 65370**	C_1019_H_1668_N_278_O_359_S_3_	3327	61.44	49		326	10	36098.18	scaffold3528.1	chlo	S	sp
**CotAD_ 66245**	C_4148_H_6934_N_1360_O_1732_S_337_	14511	45.38	25.26	0.792	450	5	48836.2	Dt08_chr24	chlo	C	sp
**CotAD_ 66538**	C_1991_H_3337_N_643_O_823_S_168_	6962	59.16	26.99	0.866	211	10	23424.96	At_chr04	chlo	S	sp
**CotAD_ 66551**	C_2086_H_3485_N_685_O_872_S_114_	7242	18.66	32.8	0.72	225	9	25226.24	scaffold3976.1	cyto	_	sp
**CotAD_ 66774**	C_1993_H_3326_N_658_O_830_S_137_	6944	42.93	29.27	0.768	216	10	24090.84	Dt08_chr24	chlo	S	sp
**CotAD_ 66775**	C_2066_H_3445_N_685_O_872_S_139_	7207	32.12	26.21	0.682	225	10	25078.29	Dt08_chr24	chlo	_	other
**CotAD_ 67823**	C_2035_H_3392_N_676_O_841_S_191_	7135	53.53	23.44	0.861	222	10	23928.26	At_chr08	cyto	S	sp
**CotAD_ 68063**	C_2031_H_3396_N_664_O_856_S_167_	7114	50.86	22.36	0.733	218	9	23245.72	At_chr03	cyto	_	sp
**CotAD_ 68189**	C_1936_H_3242_N_628_O_808_S_135_	6749	44.73	28.91	0.772	206	7	22579.21	At_chr10	chlo	S	sp
**CotAD_ 69737**	C_1966_H_3281_N_649_O_821_S_117_	6834	32.83	31.38	0.732	213	10	23867.69	scaffold2095.1	chlo	S	sp
**CotAD_ 69738**	C_1956_H_3270_N_640_O_824_S_101_	6791	32.31	31.82	0.669	210	10	23893.04	scaffold2095.1	chlo	S	sp
**CotAD_ 70003**	C_1807_H_3029_N_583_O_761_S_120_	6300	6.91	27.71	0.713	191	10	20942.44	At_chr12	cyto	_	sp
**CotAD_ 70190**	C_3927_H_6552_N_1300_O_1658_S_217_	13654	30.66	30.05	0.661	430	5	48185.02	scaffold4817.1	cyto	_	other
**CotAD_ 70192**	C_1226_H_2050_N_400_O_509_S_77_	4262	34.45	31.91	0.776	130	5	14420.49	scaffold4817.1	nucl	C	other
**CotAD_ 71431**	C_1743_H_2916_N_568_O_719_S_152_	6098	46.46	26.33	0.874	186	10	20579.98	Dt05_chr19	extr	C	sp
**CotAD_ 72458**	C_1788_H_2988_N_586_O_760_S_119_	6241	39.96	24.83	0.644	192	10	20613.31	scaffold3083.1	cysk	_	sp
**CotAD_ 72913**	C_2901_H_4845_N_955_O_1214_S_173_	10088	38.37	31.06	0.726	315	5	35071.89	scaffold4398.1	cysk	_	other
**CotAD_ 73966**	C_2955_H_4938_N_970_O_1228_S_230_	10321	41.06	27.38	0.809	320	10	35484.73	At_chr12	chlo	S	sp
**CotAD_ 74713**	C_1998_H_3351_N_643_O_829_S_165_	6986	56.41	26.68	0.843	211	9	23479.93	Dt08_chr24	golg	S	sp
**CotAD_ 76129**	C_1937_H_3235_N_637_O_793_S_190_	6792	54.41	24.72	0.935	209	10	23626.51	At_chr12	chlo	_	sp

The cell compartmentalization of stress related genes is fundamental to their functional role (Osman *et al.* 2009), the presence of the proteins encoding *LEA2* genes in the chloroplast, could be responsible for maintaining osmotic balance and suppression of reactive oxygen species (ROS) production in the guard cells (Wang *et al.* 2016), while those present in the membrane, could be responsible for the protection of the membrane integrity (Guo *et al.* 2009). In addition, the sub cellular localized proteins encoding *LEA2* genes embedded in the channeling or transporter organelles such endoplasmic reticulum, are likely to aid in the process of the ions sequestration (Porcel *et al.* 2005). Based on various findings, the LEA protein families are known to have a universal structure, with varying proportions of the various amino acids (Hong-bo *et al.* 2005). In order to verify the LEA2 proteins due to their unique hydrophobic property, we found that the LEA2s are rich in non-polar aliphatic amino acid residues, in which the highest proportion was noted in leucine with 9.2%, Valine with 8.2%, isoleucine (6.3%), alanine (5.9%) and the least was proline (5.7%). The high proportions of the non-polar residues, indicated that the LEA2 proteins are mainly embedded within the membrane, non-polar amino acids are found in the center of water soluble proteins while the polar amino acids are found at the surface (Petukhov *et al.* 1998). The second in proportions were the polar, non-charged residues such as serine (8.9%), threonine (6.4%), cysteine 1.9%), methionine (2.2%), asparagine (5.0%) and glutamine (3.4%) The high proportions of the polar residues have been found to be predominant among the stress related proteins, such as the heat shock proteins (*HSPs*) (Wang *et al.* 2004), therefore the presence of the polar residue, indicated that the LEA2 proteins could be responsible for coating the cellular macromolecules with a cohesive water layer and in turn protect the membrane and the membrane bounds multiprotein complexes from unfolding and aggregation during drought stress condition.

### Genomic organization and motif detection of LEA2 proteins in cotton

Analysis of the exon-intron structure of all the 157 *LEA2* genes was done using the gene structure displayer (http://gsds.cbi.pku.edu.cn/), a greater percentage of the *LEA2* genes and their exons were highly conserved within the group (Supplementary Figure S1). Most of the *LEA2* genes were intronless, with 114 genes, accounting for over 73%, of the LEA2s found to be intronless. The existence of introns in a genome is argued to cause enormous burden on the host (Wahl *et al.* 2009). The burden is because the introns requires a spliceosome, which is among the largest molecular complexes in the cell, comprising of 5 small nuclear RNAs and more than 150 proteins (Wahl *et al.* 2009). Intron transcription is costly in terms of time and energy (Lane and Martin 2010). Due to various stresses in which the plants are exposed to, the energy demand for survival is relatively high, thus various gene actions within the plant has to function under conserved energy demand threshold (Timperio *et al.* 2008). A plant under stress condition requires to survive the effects caused by overload of excessive production of reactive oxygen species (ROS), 3,4-Methylenedioxyamphetamine (MDA) and low levels of Peroxidase (PODs) activities, therefore most of the genes responsible for stress tolerance either lack introns or possess significantly reduced number of introns within their gene structure (Jeffares *et al.* 2008). Being the transcription process of the intron laden genes requires a lot of time and energy, which is hypothesized to cause or results into deleterious effect on gene expression (Calderwood *et al.* 2003). Conserved motifs in the 157 LEA2 proteins were identified through an online tool MEME (Supplementary Figure S1). The motif lengths identified by MEME (http://meme-suite.org/), were between 14 and 112 amino acids in LEA2 proteins, similar results of conserved motif with lengths between 11 and 164 amino acids were obtained in cotton MYBs protein (He *et al.* 2016). The homology in motif lengths with that of MYBs provided significant evidence supporting the possible role of the LEA2s in response to water stress which includes the regulation of stomatal movement, the control of suberin and cuticular waxes synthesis and the regulation of flower development (He *et al.* 2016). Most of the LEA2 proteins had distinctive motifs, which are valuable for their identification, the common motifs identified for the cotton LEA proteins were; motif 1 (FFVLFSVFSLILWGASRPQKPKITMKSIKFENFKIQAGSDFSGVPTDMITMNSTVKMTYRNTATFFGVHVTSTPLDLSYSQJTIASG), motif 2 (WLVFRPKKPKFSLQSVTVYAL), motif 3 (NFQVTVTARNPNKRIG IYYD), motif 5 (TVKNPNFGSFKYDNSTVSVNYRGKVVGEA) and motif 14 (RRRSCCCCCCLWTLJ) (Supplementary Figure S2).

The number of the conserved motifs in each LEA2s varied between 1 and 7. The majority of close members in the phylogenetic tree exhibited common motif compositions, which suggested they have a functional similarity within the same subgroup. The alignment results of the LEA2 proteins showed various segments such as Y-segment, K-segment and S-segments (Supplementary Figure S3), which have been previously described in dehydrins (Hanin *et al.* 2011). Other unique segments identified were E, R and D segments. The K segment has been found to form an amphipathic α-helix (Monera *et al.* 1995). The K-segments assumes α-helical structure identical to class A2 amphipathic α-helices mainly found in apolipoproteins, apolipoproteins facilitate the transportation of water-insoluble lipids in plasma, and α-synucleins (Rorat 2006). The conformation of the protein structure in turn leads to functional change (Dyson and Wright 2005). Drought stress alters the protein ambient microenvironment, leading to protein conformational and functional changes (Mahdieh *et al.* 2008). The amphipathic α-helices have the ability to interact with the dehydrated surfaces of various other proteins and biomembranes (Cornell and Taneva 2006). The binding of dehydrins to the dehydrated surface of other proteins enhances formation of amphipathic α-helices which protects other proteins from further loss of water. The presence of this K segment in LEA2 revealed the significant role played by these proteins in plants during drought stress. It has been suggested that the protective role of the LEA proteins is due to their ability to form α-helices which enables them to interact with other proteins and or biomembranes (Koag 2003). Kovacs *et al.*, (Kovacs *et al.* 2008), reported the protective activities of two dehydrin proteins isolated from *A. thaliana*, early response to dehydration 10 (*ERD10)* and early response to dehydration 14 (*ERD14)*, against thermal inactivation of alcohol dehydrogenase and thermal aggregation of citrate synthase.

### Chromosomal location and duplication events of cotton *LEA2* genes

A gene’s location on a chromosome plays a significant role in shaping how an organism’s traits vary and evolve (Lazazzera and Hughes 2015). Chromosomes hold thousands of genes, with some situated in the middle of their linear structure and others at either end (Bickmore and Van Steensel 2013). Therefore, for us to understand the gene distribution and mapping positions of the *LEA2* genes, the positions of each *LEA2* genes were mapped on the A, D and AD cotton chromosome by carrying out homology search against the full-lengths of *G. arboreum* (A-genome), *G. raimondii* (D-genome) and *G. hirsutum* (AD genome) assembly. The *LEA2* genes were mapped in all the 26 chromosomes in *G. hirsutum*, 13 chromosomes in *G. arboreum* and 12 chromosomes in *G.raimondii*. In diploid cotton genome, *G. arboreum* and *G. raimondii*, the gene distribution pattern was almost identical to the tetraploid cotton gene distribution (Supplementary Figure S4). In chromosome 9 in *G. arboreum* and its homolog chromosome in *G. raimondii*, a significant level of gene loss was observed in which only a single gene was contained in chr09 of *G. arboreum* compared to 10 genes in chr09. But more interestingly, there was total gene loss in chr13 of *G. raimondii*. The lack of *LEA2* genes in chr13 in *G. raimondii* could only be accounted for due to either gene loss or gene deletion, for most of the *LEA* genes are found in every chromosome. The occurrences of *LEA2* genes on every chromosome indicated that the genes are widely distribution on the entire cotton genome. However, the density of these loci was variable across the 26 chromosomes of upland and 13 chromosomes in A and D diploid cotton. The largest number of genes were located on chromosomes At09 (chr09) and Dt09 (chr23), with 12 and 14 genes respectively, followed by chromosome, Dt08 (chr24) with 10 genes, Dt 06 (chr25) with 9 genes, At07 and At12 with 12 genes each. The lowest loci ranged from 1 to 5 genes, with chromosome At02, At05, At09, Dt02 (chr14) and Dt04 (chr22) had a single gene each (Supplementary Figure S5). A total of 39 genes were not mapped and thus grouped as scaffold. The distribution of the genes on the chromosomes appeared to be uneven.

In general, the central sections of chromosomes were located with less *LEA2* genes and relatively high densities of upland cotton LEA2s were observed in the top and bottom sections of most chromosomes. Similar gene loci clustering pattern was also observed in *GrMYB* genes distribution in which most of the genes were clumped either on the upper or lower regions of the chromosomes (He *et al.* 2016). A gene’s location on a chromosome plays a significant role in shaping how an organism’s traits vary and evolve (Sexton and Cavalli 2015). It has been found that evolution is less a function of what a physical trait is, but more of where the genes that affect that trait are located in the genome (Sexton and Cavalli 2015). The distribution of this subset of *LEA* genes across the whole cotton genome provided a significant role played by these genes within the plant.

The main cause of gene expansion in a genome or organism is either due to segmental or tandem duplication (Cannon *et al.* 2004). Two or more genes located on the same chromosome, one following the other, confirms a tandem duplication event, while gene duplication on different chromosomes is designated as segmental duplication event (Yu *et al.* 2005). In the present study, cluster formations by the *LEA2* genes explained the mechanism behind their expansion in cotton. Most of the duplicated genes were between *G. hirsutum* and its ancestors, *G. arboreum* (53) and *G. raimondii* (11) (Table 3). The tetraploid cotton, *G. hirsutum* evolved due to whole genome duplication resulting into polyploidy cotton. The Ka/Ks values ranged from 0 to 2.17333, with an average value of 0.4238, which implied that majority of the gene pair had Ka/Ks values of less than 1, which indicated that the *LEA*2 genes have been influenced extensively by purifying selection during the process of their evolution.

**Table 3:** Gene duplication, synonymous (Ks), nonsynonymous (Ka) and Ka/Ks values calculated for paralogous *LEA2* gene pairs in cotton genome

Gene type	Paralogous gene pairs		Length (aa)	Ka	Ks	Ka/Ks	Negative/purifying selection	P-Value (Fisher)
**LEA2**	CotAD_59405	CotAD_76129	627	0	0.00654	0	YES	0
**LEA2**	CotAD_20020	Cotton_A_01845	750	0	0.00568	0	YES	0
**LEA2**	CotAD_19078	Cotton_A_23172	648	0	0.00672	0	YES	0
**LEA2**	CotAD_08181	Cotton_A_27543	606	0	0.00697	0	YES	0
**LEA2**	CotAD_48976	Cotton_A_29779	660	0	0.00642	0	YES	0
**LEA2**	CotAD_35514	Gorai.010G176400.1	543	0	0.00822	0	YES	0
**LEA2**	CotAD_31536	Cotton_A_13470	627	0.00211	0.03373	0.06246	YES	0.00360292
**LEA2**	CotAD_37888	Cotton_A_08663	960	0.04378	0.55839	0.07841	YES	1.73E-37
**LEA2**	CotAD_03649	CotAD_37888	960	0.04522	0.54142	0.08352	YES	9.32E-36
**LEA2**	CotAD_03649	Cotton_A_14478	960	0.04592	0.52972	0.08668	YES	3.29E-35
**LEA2**	CotAD_03649	CotAD_73966	960	0.04597	0.527	0.08723	YES	4.70E-35
**LEA2**	CotAD_17102	CotAD_31536	627	0.00422	0.03365	0.12547	YES	0.0107355
**LEA2**	CotAD_44941	Gorai.005G203000.1	720	0.00175	0.01368	0.12779	YES	0.0998325
**LEA2**	CotAD_08181	CotAD_46550	606	0.00654	0.04975	0.1315	YES	0.00250188
**LEA2**	CotAD_17101	Cotton_A_13469	666	0.00195	0.01318	0.14805	YES	0.121749
**LEA2**	CotAD_09578	Cotton_A_02196	780	0.0903	0.59944	0.15064	YES	7.07E-24
**LEA2**	CotAD_35069	CotAD_62996	954	0.00551	0.03643	0.15116	YES	0.0017334
**LEA2**	CotAD_59405	Cotton_A_40363	627	0.00636	0.04016	0.15842	YES	0.00848415
**LEA2**	CotAD_17045	Cotton_A_14354	657	0.00201	0.01262	0.15958	YES	0.13409
**LEA2**	CotAD_09685	CotAD_53981	753	0.00711	0.04386	0.16211	YES	0.00252472
**LEA2**	CotAD_01700	Cotton_A_02196	780	0.09992	0.58986	0.16939	YES	8.68E-22
**LEA2**	CotAD_17062	CotAD_21731	732	0.00719	0.04161	0.17276	YES	0.00506705
**LEA2**	CotAD_35069	Cotton_A_24356	954	0.00551	0.03178	0.17329	YES	0.00508945
**LEA2**	CotAD_10376	Cotton_A_05625	831	0.00645	0.03444	0.18723	YES	0.00723285
**LEA2**	CotAD_21924	Cotton_A_18919	786	0.01028	0.05219	0.19697	YES	0.00026749
**LEA2**	CotAD_31535	Gorai.006G150200.1	666	0.00391	0.01981	0.19743	YES	0.082505
**LEA2**	CotAD_25271	Cotton_A_14676	405	0.00647	0.03234	0.20023	YES	0.085476
**LEA2**	CotAD_09685	Cotton_A_05444	753	0.0089	0.04387	0.20282	YES	0.00516244
**LEA2**	CotAD_46888	Cotton_A_09596	573	0.00922	0.0453	0.20351	YES	0.0147038
**LEA2**	CotAD_08181	Gorai.009G305100.1	606	0.00435	0.02103	0.20672	YES	0.090366
**LEA2**	CotAD_19078	CotAD_66774	648	0.01009	0.04842	0.20844	YES	0.00834864
**LEA2**	CotAD_32487	Cotton_A_13240	630	0.00425	0.01917	0.22185	YES	0.103356
**LEA2**	CotAD_23118	CotAD_74061	1215	0.01611	0.06882	0.23405	YES	5.00E-05
**LEA2**	CotAD_36328	CotAD_64346	630	0.01777	0.07564	0.23489	YES	0.000973496
**LEA2**	CotAD_32847	CotAD_39064	612	0.01106	0.0461	0.23994	YES	0.0153075
**LEA2**	CotAD_46873	CotAD_60617	630	0.00835	0.03452	0.24185	YES	0.0372109
**LEA2**	CotAD_46873	Cotton_A_09615	630	0.00835	0.03452	0.24185	YES	0.0372109
**LEA2**	CotAD_18546	CotAD_37776	519	0.01016	0.04195	0.24212	YES	0.0375368
**LEA2**	CotAD_19375	Cotton_A_06435	675	0.01345	0.05541	0.24268	YES	0.00759106
**LEA2**	CotAD_46888	CotAD_61391	573	0.01387	0.05313	0.26111	YES	0.0175133
**LEA2**	CotAD_23118	Cotton_A_38117	1215	0.01611	0.06077	0.26514	YES	0.000321992
**LEA2**	CotAD_19214	Cotton_A_30889	543	0.00237	0.0083	0.28598	YES	0.347253
**LEA2**	CotAD_31535	Cotton_A_13469	666	0.01377	0.04718	0.2919	YES	0.0234164
**LEA2**	CotAD_21924	CotAD_64657	786	0.01373	0.04693	0.29247	YES	0.0120925
**LEA2**	CotAD_31140	Cotton_A_15998	747	0.00174	0.0058	0.30099	YES	0.356655
**LEA2**	CotAD_30219	Cotton_A_32495	597	0.01105	0.03626	0.30482	YES	0.0618481
**LEA2**	CotAD_46873	Gorai.001G124400.1	630	0.00208	0.00674	0.30909	YES	0.361889
**LEA2**	CotAD_46888	Gorai.001G122700.1	573	0.0046	0.0148	0.31039	YES	0.238274
**LEA2**	CotAD_28252	CotAD_53263	492	0.01356	0.04285	0.31656	YES	0.069282
**LEA2**	CotAD_14147	Cotton_A_02370	636	0.00416	0.01312	0.3169	YES	0.244174
**LEA2**	CotAD_23646	Cotton_A_27300	609	0.04249	0.13135	0.32348	YES	0.000630664
**LEA2**	CotAD_09578	Cotton_A_07036	780	0.00342	0.01037	0.33004	YES	0.256013
**LEA2**	CotAD_17045	CotAD_64004	657	0.02247	0.06523	0.34445	YES	0.0157104
**LEA2**	CotAD_37888	CotAD_73966	960	0.01528	0.0442	0.34576	YES	0.0157353
**LEA2**	CotAD_37888	Cotton_A_14478	960	0.01247	0.03528	0.3534	YES	0.0321315
**LEA2**	CotAD_23646	Gorai.006G199800.1	609	0.04249	0.11411	0.37237	YES	0.00460089
**LEA2**	CotAD_17062	Cotton_A_14370	732	0.0099	0.02648	0.37402	YES	0.0618224
**LEA2**	CotAD_02652	Cotton_A_02370	636	0.01256	0.03311	0.37934	YES	0.101339
**LEA2**	CotAD_19214	CotAD_35514	543	0.00955	0.02509	0.38065	YES	0.19023
**LEA2**	CotAD_21731	Cotton_A_14370	732	0.00899	0.02354	0.38192	YES	0.138838
**LEA2**	CotAD_13584	Cotton_A_01845	750	0.00878	0.02294	0.38264	YES	0.139381
**LEA2**	CotAD_17101	CotAD_31535	666	0.01576	0.04026	0.3915	YES	0.077316
**LEA2**	CotAD_35091	CotAD_60435	753	0.03016	0.07689	0.39221	YES	0.0144267
**LEA2**	CotAD_20308	CotAD_70003	573	0.00915	0.02291	0.39918	YES	0.206152
**LEA2**	CotAD_50359	CotAD_66538	633	0.01677	0.04094	0.40958	YES	0.0891624
**LEA2**	CotAD_01700	Cotton_A_07036	780	0.01551	0.03701	0.41916	YES	0.0752732
**LEA2**	CotAD_02652	CotAD_14147	636	0.00835	0.01974	0.42281	YES	0.226532
**LEA2**	CotAD_35513	Cotton_A_30890	651	0.02193	0.05102	0.42978	YES	0.0738291
**LEA2**	CotAD_35514	Cotton_A_30889	543	0.00716	0.01659	0.43135	YES	0.312651
**LEA2**	CotAD_28872	Gorai.005G203000.1	720	0.01233	0.02762	0.44641	YES	0.170613
**LEA2**	CotAD_56699	Cotton_A_38534	639	0.02021	0.04493	0.44988	YES	0.106618
**LEA2**	CotAD_01700	CotAD_09578	780	0.01202	0.02634	0.45642	YES	0.151105
**LEA2**	CotAD_40972	Cotton_A_29659	591	0.96659	2.0709	0.46675	YES	0.00123143
**LEA2**	CotAD_40972	CotAD_38978	591	0.96025	2.04193	0.47026	YES	0.00125135
**LEA2**	CotAD_17101	Gorai.006G150200.1	666	0.01977	0.04018	0.49197	YES	0.209339
**LEA2**	CotAD_50359	Cotton_A_33548	633	0.01678	0.03388	0.49528	YES	0.175709
**LEA2**	CotAD_74713	Cotton_A_33548	633	0.01678	0.03388	0.49528	YES	0.175709
**LEA2**	CotAD_03649	CotAD_31344	960	0.01103	0.02214	0.49798	YES	0.177084
**LEA2**	CotAD_13584	CotAD_20020	750	0.00878	0.01711	0.51348	YES	0.287642
**LEA2**	CotAD_13115	Cotton_A_31059	576	0.0207	0.0379	0.54625	YES	0.312514
**LEA2**	CotAD_19214	Gorai.010G176400.1	543	0.00955	0.01664	0.57418	YES	0.403293
**LEA2**	CotAD_20308	Cotton_A_17625	573	0.01375	0.02296	0.59881	YES	0.347235
**LEA2**	CotAD_25271	CotAD_48769	405	0.00647	0.01063	0.6094	YES	0.539117
**LEA2**	CotAD_12681	Cotton_A_08212	432	0.03121	0.04928	0.63319	YES	0.35887
**LEA2**	CotAD_19623	CotAD_36999	282	0.03296	0.04771	0.69081	YES	0.631725
**LEA2**	CotAD_23646	Cotton_A_27282	609	0.02587	0.03738	0.69204	YES	0.542393
**LEA2**	Cotton_A_13471	CotAD_17103	180.94	2.32397	1.11323	0.77822	YES	837
**LEA2**	CotAD_53438	CotAD_68189	618	0.02341	0.02898	0.80786	YES	0.519399
**LEA2**	CotAD_56696	Cotton_A_38535	630	0.01838	0.02269	0.80979	YES	0.670475
**LEA2**	CotAD_44941	Cotton_A_17986	720	0.01233	0.01369	0.90056	YES	0.874489
**LEA2**	CotAD_22539	Cotton_A_25195	408	1.23265	1.24112	0.99317	YES	1
**LEA2**	CotAD_28872	CotAD_44941	720	0.0141	0.01369	1.03042	NO	0.900519
**LEA2**	CotAD_17103	Cotton_A_13471	837	2.58712	2.32397	1.11323	NO	0.778217
**LEA2**	CotAD_13827	Cotton_A_18645	1104	2.12092	1.89653	1.11832	NO	0.642563
**LEA2**	CotAD_10044	Cotton_A_09473	1902	0.00274	0.00228	1.20458	NO	0.731531
**LEA2**	Cotton_A_31083	CotAD_35069	939	2.2748	1.83858	1.23726	NO	0.447623
**LEA2**	CotAD_30219	Gorai.006G104100.1	597	0.00884	0.00707	1.25015	NO	0.743557
**LEA2**	CotAD_03649	Cotton_A_08663	960	0.00549	0.00437	1.25606	NO	0.744588
**LEA2**	CotAD_11658	Cotton_A_40499	789	0.02309	0.01751	1.3191	NO	0.985982
**LEA2**	CotAD_12375	CotAD_42408	597	2.42062	1.68288	1.43838	NO	0.288342
**LEA2**	CotAD_35091	Cotton_A_24371	699	3.50309	1.61186	2.17333	NO	0.036477

### Cis element prediction in LEA2 proteins

**Figure 2:**
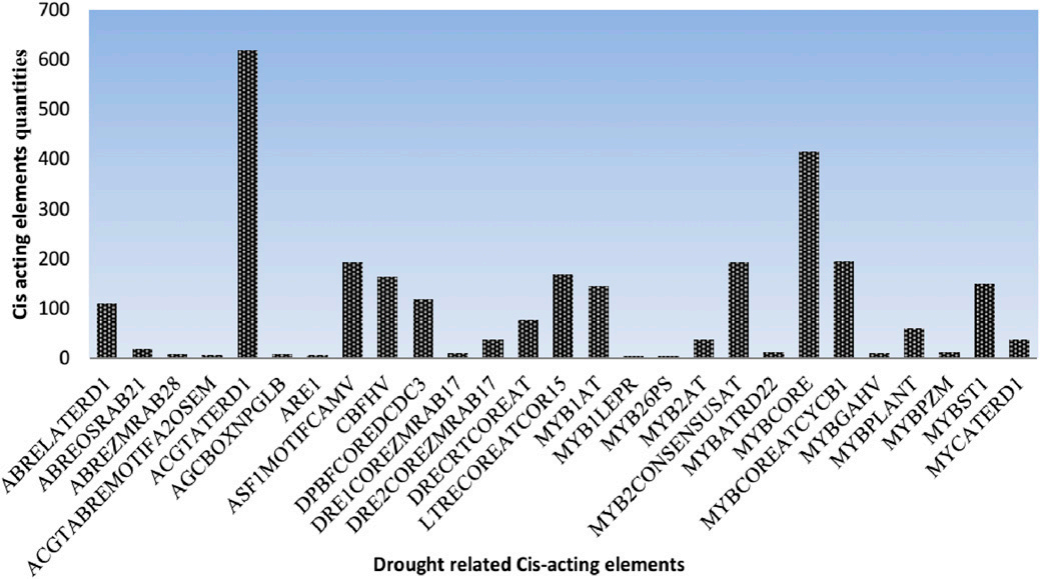
Average number of the *cis*-elements in promoter region of upland cotton *G. hirsutum LEA*2 genes. The *cis*-elements were analyzed in the 1 kb upstream promoter region of translation start site using the PLACE database.

Transcription factors (TFs) and *cis*-acting regulatory elements contained in stress-responsive promoter regions function not only as molecular switches for gene expression, but also as terminal points of signal transduction in the signaling processes *(**Chang et al. 2008**). The cis*-regulatory promoters are located on the upstream of genes and functions as binding sites for transcription factors (TFs) which play essential functions in determining the tissue-specificity or stress-responsive expression patterns of the genes (Yamaguchi-Shinozaki and Shinozaki 2005). For better understanding of the potential roles of the *LEA2* genes, 1000 bp regions upstream of the transcriptional start site were extracted and used in the identification of *cis*-regulatory promoters and other important motifs. Abiotic stress-related *cis*-elements were found in the putative promoters of *LEA2* genes in *upland cotton*, *G. hirsutum*, (Figure 2) and (Supplementary table S5). For instance, MYBCORE, is known to have a functional role in drought and regulation of flavonoid biosynthesis (Solano *et al.* 1995). ABRELATERD1, ABRE-like sequence and ACGTATERD1 are responsive to dehydration (Simpson *et al.* 2003). ACGTATERD1 is associated to early responsive to dehydration (Simpson *et al.* 2003). The presence of the stress promoter elements strongly supported the possible role of upland cotton LEA2 proteins in enhancing drought tolerance in cotton. The high proportion of cis promoter elements in LEA2 proteins, could possibly explain why genes encoding LEA proteins are highly expressed under abiotic stress, as was found in the root tissues of *Arabidopsis* under drought stress (Dalal *et al.* 2009; Candat *et al.* 2014). It is also important to mention that various transcription factors (TFs) and *cis*-acting regulatory elements contained in stress-responsive promoter regions function not only as molecular switches for gene expression, but also as terminal points of signal transduction in the signaling processes (Yamaguchi-Shinozaki and Shinozaki 2005).

### Prediction of *LEA* genes targeted by miRNAs

Drought is a recurring climate feature in most parts of the world (Kang *et al.* 2009). The sessile nature of the plants, has made the plants to developed their own defense systems to cope up with perennial and erratic adverse climatic conditions (Bartwal *et al.* 2013). One of the defense mechanisms used by the plants toward the effect of drought stress is the reprogramming of gene expression by microRNAs (Ferdous *et al.* 2015). The small RNAs (miRNAs) are known as the small noncoding RNAs with approximately 22 nucleotides length. The miRNAs are mainly involved in the regulation of genes at post-transcriptional levels in a range of organisms (Grivna *et al.* 2006). Large groups of small RNAs have been reported as regulators in plant adaptation to abiotic stresses (Xie *et al.* 2015). To get more information on the *LEA2* genes functions, we determined the prediction of miRNAs targets on *LEA2* genes by the use of psRNATarget, the same as been applied for other functional genes in cotton (Dai and Zhao 2011). Out of 157 upland cotton *LEA2* genes, 63 genes were found to be targeted by 48 miRNAs, representing 40% of all the *LEA2* genes (Supplementary Table S6). The highest levels of target was detected for the following genes with more than 6 miRNAs, *CotAD_00799* being targeted by ghr-miR2948-5p, ghr-miR7492a, ghr-miR7492b, ghr-miR7492c, ghr-miR7494 and ghr-miR7510b. *CotAD_19205* targeted by ghr-miR390a, ghr-miR390b, ghr-miR390c, ghr-miR7492a, ghr-miR7492b and ghr-miR7492c. *CotAD_31936* targeted by ghr-miR7492a, ghr-miR7492b, ghr-miR7492c, ghr-miR827a, ghr-miR827b and ghr-miR827c. *CotAD_32487* targeted by ghr-miR156a, ghr-miR156b, ghr-miR156d, ghr-miR7507 and ghr-miR7509. *CotAD_33143* targeted by ghr-miR2948-5p, ghr-miR482a, ghr-miR7492a, ghr-miR7492b, ghr-miR7492c and ghr-miR7510b. *CotAD_41925* targeted ghr-miR396a, ghr-miR396b, ghr-miR7492a, ghr-miR7492b, ghr-miR7492c, ghr-miR827a, ghr-miR827b and ghr-miR827c. The rest of the genes were either targeted by 1 or 5 miRNAs. The high number of miRNAs targeting *LEA2* genes could possibly have direct or indirect correlation to their stress tolerance levels to abiotic stress more so drought. Some specific miRNAs had high level of target to various genes such as ghr-miR164 (4 genes), ghr-miR2949a-3p (4 genes), ghr-miR2950 (8 genes), ghr-miR7492a (10 genes), ghr-miR7492b (10 genes), ghr-miR7492c (10 genes), ghr-miR7504a (5 genes), ghr-miR7507 (5 genes), ghr-miR7510a (6 genes), ghr-miR7510b (10 genes), ghr-miR827b (4 genes) and lastly ghr-miR827c (4 genes). It has been found that miRNAs might be playing a role in response to drought and salinity stresses through targeting a series of stress-related genes.

The plant specific transcriptome factors such as *NAC* gene family have been found to have varied functional roles in plant growth and development (Pereira-Santana *et al.* 2015), myeloblastosis (MYB) is highly correlated to various stress factors (Ambawat *et al.* 2013). The detection of some the LEA2 genes being targeted by specific miRNA linked to mitogen-activated protein kinase (*MAPK*), N-acetyl-L-cysteine (*NAC*) and myeloblastosis (*MYB*) provided a stronger indication of the significance contributions of the LEA2s in enhancing drought tolerance in plants. The micro/small RNAs mediated post-transcriptional processes have been linked to response to water deficit condition. Plant miRNAs are involved in multi-complex and arrays of processes, including but not limited to response to stress, nutrient limitation, development, pattern formation, flowering time, hormone regulation, and even self-regulation of the miRNA biogenesis pathway (Yamaguchi-Shinozaki and Shinozaki 2005). It is important to note that most of the miRNA target genes encode transcription factors, which place miRNAs at the focal point of gene regulatory networks. Moreover, the availability of genome-wide characterization of cotton miRNA genes enabled us to perform the prediction of the miRNA targets involved in drought response.

### Expression Patterns of *LEA2* Genes in Different Tissues of Upland cotton as determined Through RNA sequence

**Figure 3:**
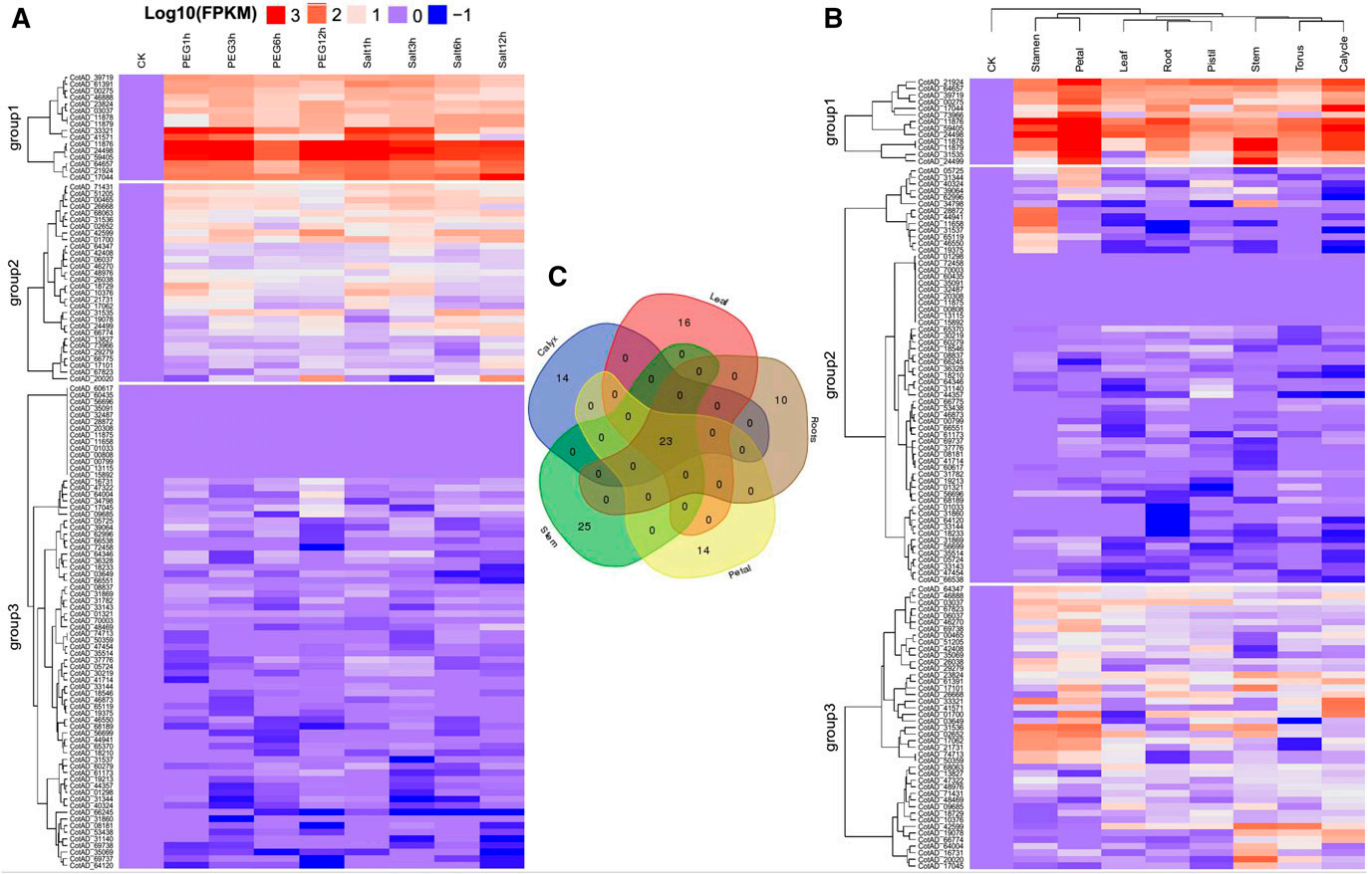
Expression profile analysis of *LEA2* genes in 5 upland cotton tissues. The *LEA2* genes expressed (RPKM > 1) in leaf, stem, root, calyx and petal were represented according to their tissue specificity: (A): *LEA2* genes RNA seq. expression profile under drought and salt stress. (B): LEA2 expression in the 8 different tissues and (C): Venn diagram quantification and common genes expressed among the 5 tissues.

Analysis of the RNA expression profile provides an indicator of the functional role of the genes in the plant. We therefore carried the RNA expression analysis (RPKM > 1) in various tissues of the cotton plant, out of the entire 157 *LEA2* genes in upland cotton, *G. hirsutum*, 117 (75%) of all the *LEA2* genes showed differential expression in various tissues, such as the leaves, roots, stem, petal, pistil, stamen, torus and calycle (Figure 3). Based on their expression profiling, the genes were clustered into three broad groups. Group 1 members with 29 genes were highly up regulated under drought and salt conditions. Under salt and drought stress, *CotAD_33321*, *CotAD_41571*, *CotAD_11876*, *CotAD_24498* and *CotAD_59405* showed the highest expression levels, Similarly *CotAD_11876*, *CotAD_24498* and *CotAD_59405* were equally significantly up regulated in all the tissues tested. A total of 23 genes were highly up regulated in 5 tissues, which provided a strong evidence of the functional role of the *LEA2* genes in enhancing stress tolerance in plants. Majority of the analyzed genes, showed relatively lower expression levels in the root tissues, but *CotAD_11876*, *CotAD_59405* and *CotAD_24498* exhibited significant higher expression levels, with expression values of more than 2. A unique observation was made, among the moderately up regulated genes in the roots, the genes exhibited significant up regulation in the calyx. The up regulation of these genes in the reproductive tissues could be an indication of their functional role in the fiber development process.

In the validation of the expression profile of the *LEA2* genes under drought stress condition, *CotAD_24498*, *CotAD_21924*, *CotAD_20020* and *CotAD_59405* were highly up regulated in root, stem and roots tissues under drought stress condition. However, the expression levels were much higher in *G. tomentosum* as opposed to *G. hirsutum*, suggesting that, these genes could be the key genes.

### qRT-PCR Expression profiling of the *LEA2* genes in leaf, stem and roots of upland cotton

**Figure 4:**
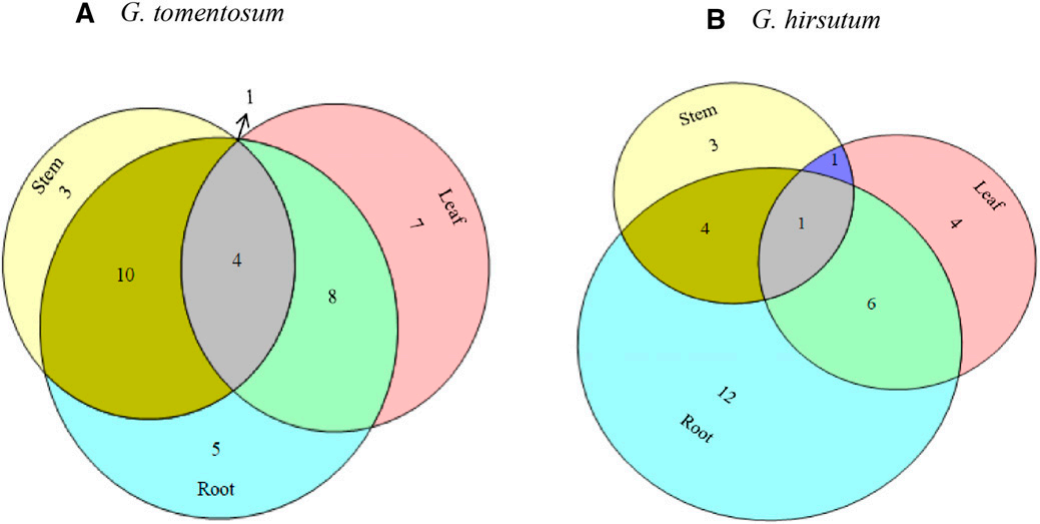
Venn den diagram of differential expressions of *LEA2* genes in different plants tissues. A. tissues of *G. hirsutum* and B. tissues of *G. tomentosum.*

Based on the results obtained from the RNA sequence data, 48 genes were selected for qRT-PCR validation. Two cotton genotypes were used, *G hirsutum* an elite cultivar, majorly grown around the world; it covers more than 90% the cotton growing regions in China but susceptible to drought stress condition. The second plant used was the *G. tomentosum*, wild cotton, native to the Hawaiian island, it is known for its high ability to tolerate salinity and drought stress conditions. The two cotton plants were grown in the greenhouse, and at three leaf stage, were exposed to drought for a period of 14 days. The roots stem and leaves were obtained for RNA extraction and qRT-PCR analysis. In the analysis of qRT-PCR profiling of various tissues, the results indicated high variability in transcript abundance of *LEA2* genes in upland cotton (Figure 4). In *G. tomentosum* and *G. hirsutum*, majority of these genes showed relatively high expression in the root and leaf, except in stem. Leaves and roots are the main plant organs affected by drought stress (Alexandersson *et al.* 2005). The plant leaf is the site for photosynthesis; drought stress might possibly be the cause of excess release of reactive oxygen species (ROS). ROS are toxic to the plants, the genes with high expression in the leaves, could perhaps be involved in the ubiquitin of the ROS, thus preventing the damage and maintain the normal functions of the photosynthetic cells. The high osmotic potential generated in the cytoplasm of guard cells during stomatal opening could probably lead to accumulation of LEA2s in leaf tissue. Increased osmotic potential within the guard cells necessitates mass flow of water into the guard cells, leading to its turgidity and thus opening of the stomatal pore, but during drought stress, the osmotic potential is never offset, and thus dehydration stress on the nucleus. The LEA2s increased accumulation within the leaf tissues, could be due to maintaining structural integrity and preventing the membranes from dehydration stress. The finding is consistent to proposed functions of the *LEA* genes, which is the protective role during abiotic stresses (Nylander *et al.* 2001). The roots are the connection point between the water reservoir and the plants. High up regulation of *LEA2* genes in the roots indicated that these genes could be involved in the water balance in the roots. Increased or high up regulation of LEA2s in the roots, further augment the primary role of *LEA* genes in plants, the protective function, roots are the very first plant organs to be affected by drought stress.

### Expression profiles of *LEA2* genes Under drought treatment in *G. hirsutum* and *G. tomentosum*

**Figure 5:**
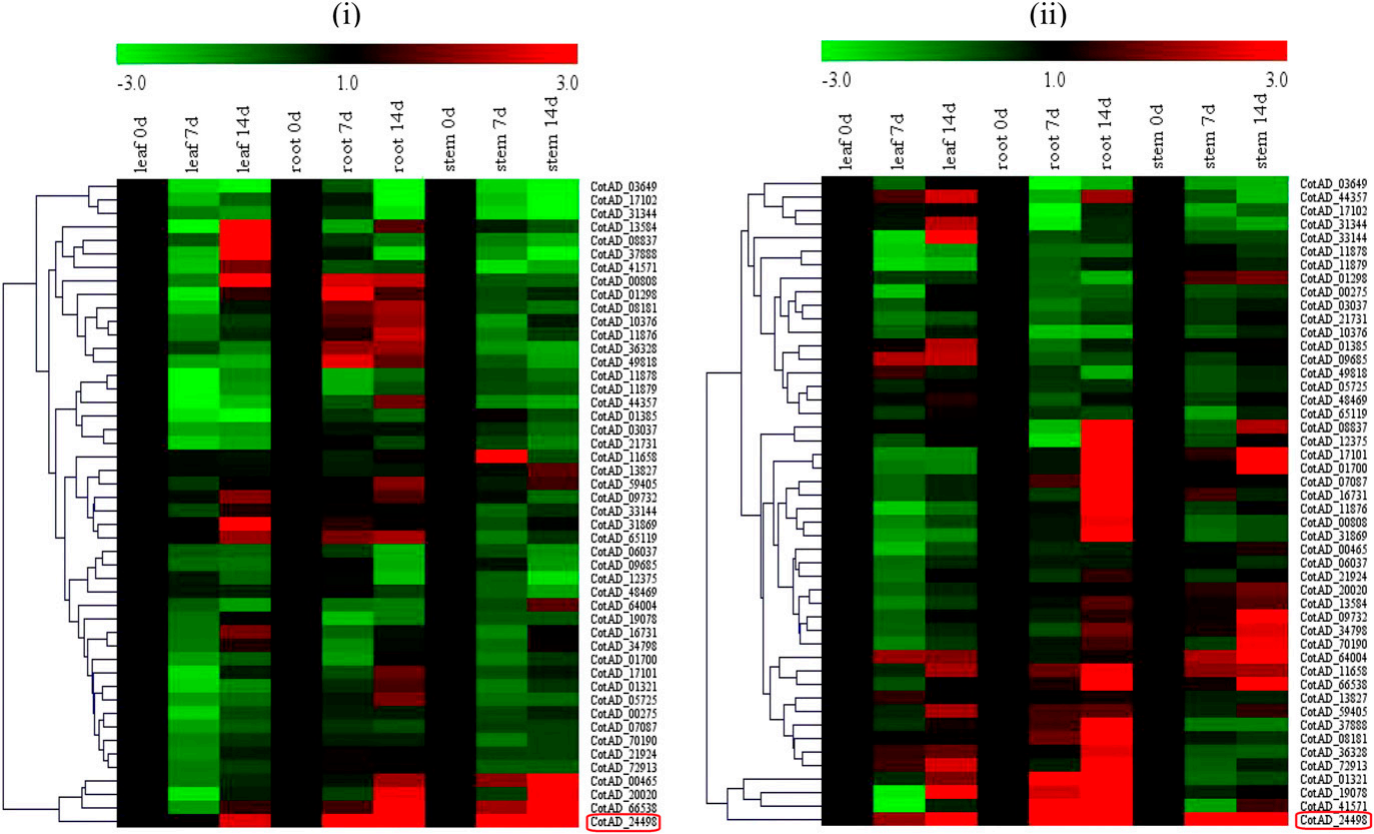
Differential expression of upland cotton *LEA2* genes under drought stress. The heat map was visualized using Mev.exe program. (Showed by log2 values) under control and in treated samples for 7 and 14 days after drought treatment (i) *G. tomentosum* and (ii) *G. hirsutum*. Red–up regulated, green-down regulated and black–no expression. Red box indicate the cloned gene.

Gene expression profile provides vital information of the roles played by the genes in plants (Movahedi *et al.* 2012). In order to determine the expression pattern of the *LEA2* genes in tolerant and non-tolerant upland cotton genotypes, we carried the qRT-PCR validation of 48 *LEA2* genes in leaves, roots and stem tissues. The 48 genes were selected based on the RNA sequence expression profile, 24 genes were up regulated while the other half were down regulated. The samples for qRT-PCR were collected at 0, 7 and 14^th^ day of stress exposure, in which 0 day (control) was used as the reference point. More genes were up regulated in all the tissues of the drought tolerant genotype, *G. tomentosum* as compared to the drought sensitive genotype, *G. hirsutum* (Figure 5). The result obtained denotes that the drought resistant genotype have the potential to mobilize more drought related genes, when exposed to drought tolerance as opposed to the less tolerant genotypes, thus the higher expression levels, similar results were obtained in the expression for cold tolerance genes in *Arabidopsis* with varying tolerance levels, more genes were up regulated in the cold tolerant and in the cold susceptible genotype (Hannah *et al.* 2006).

**Figure 6:**
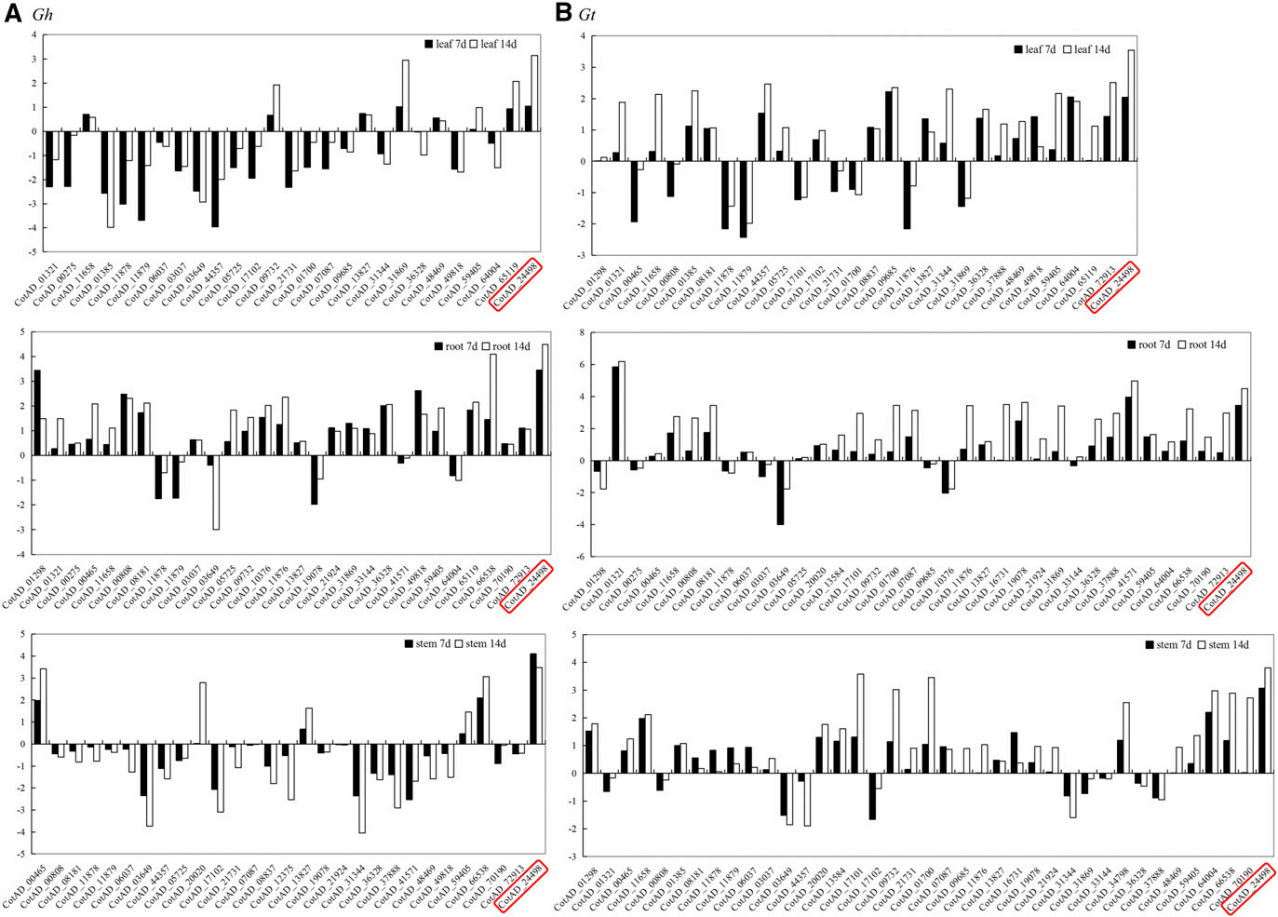
Quantitative PCR analysis of the selected *LEA2* genes. Abbreviations: 7d-7 days and 14d-14 days of stress. *Gh*–*G. hirsutum* and *Gt*–*G. tomentosum*. Y-axis: relative expression (2^-ΔΔCT^. The enclosure indicated the cloned gene.

The up regulation of *LEA2* genes under drought stress, could possibly explain their protective role in plants tissues under dehydration stress. For instance, *HVA1*, a *LEA* gene from barley (*Hordeum vulgare* L) was found to confer drought stress in transgenic rice (Babu *et al.* 2004). Interestingly, some phylogenetic *LEA2* gene pairs, orthologous genes were found to have differential expression pattern in either of the cotton genotypes (Figure 6), for instance, *CotAD_71431* and *CotAD_51205* exhibited varied expression pattern under drought and salt stress conditions as evident in the RNA expression analysis. The result suggests that even if these genes are cladded together; they could have developed different biological function over time. Orthologous genes are members of the genes with a common evolutionary origin and share greater percentage of sequence similarity (Nehrt *et al.* 2011). According to the expression pattern of *LEA2* genes in different tissues, it would be interesting to functionally characterize these genes in upland cotton, *G. hirsutum*. Majority of the *LEA2* genes showed higher expression level in leaf and root tissues, which indicated the functional conservation of the gene sub family. The variation in expression between *G. hirsutum* and *G. tomentosum* could be due to broad changes in environmental conditions, *G. tomentosum* exhibits divergence signals that are associated with directionally selected traits and are functionally related to stress responses. These results suggest that stress adaptation in *G. tomentosum* might have involved the evolution of protein-coding sequences and thus these genes can be introgressed in to elite upland cotton, in order to boost their performance in the current face of declining fresh water and precipitation.

### qRT-PCR Analysis of the Transformed Gene in Upland Cotton Tissues

**Figure 7:**
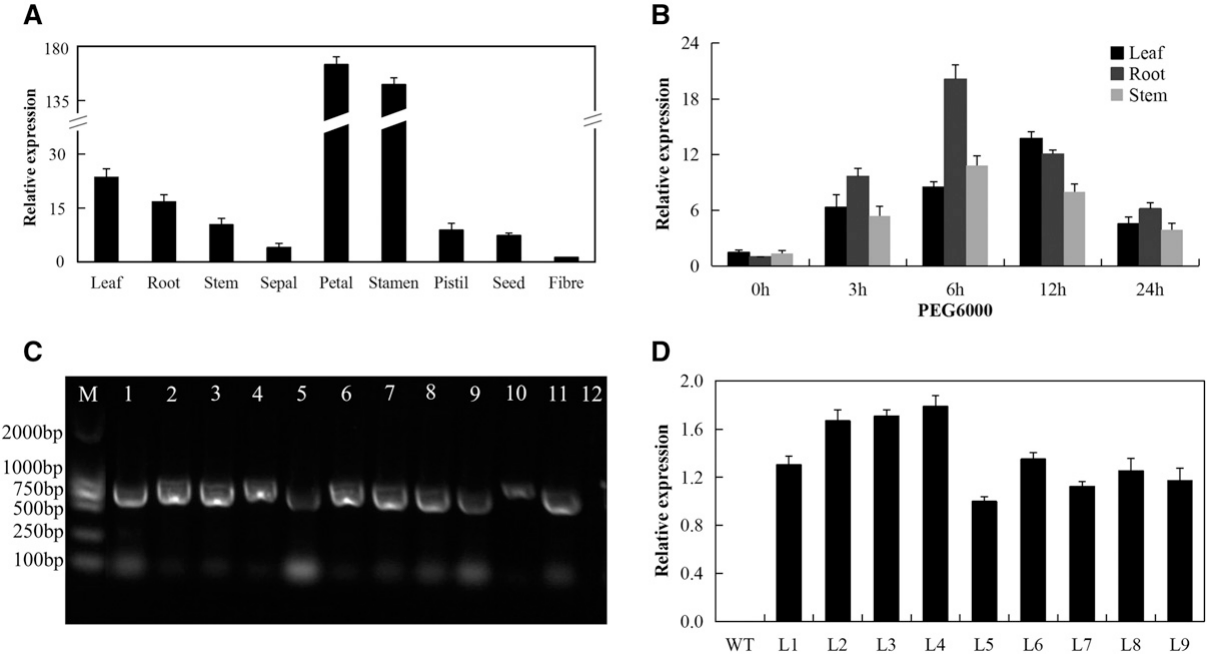
The qRT-PCR analysis of the expression of the cloned gene *CotAD_24498* (A) Total RNA isolated from various tissue of cotton plant under normal conditions; (B) Total RNA extracted from drought-stressed cotton seedlings; (C) Polymerase chain reaction (PCR) analysis performed to check 630bp coding sequence (CDS) integration in transformed T1 generation, number 1–10 transgenic lines, 11 positive control (*pWM101- CotAD_24498* and 12 is the negative control (wild-type, WT). (D) The transcripts expression levels of the *CotAD_24498* of T2 transgenic lines analyzed through qRT-PCR.

Based on the expression analysis of the *LEA2* genes in the various tissues of *G. tomentosum* (drought susceptible) and *G. hirsutum* (drought susceptible). We identified a single gene with significant expression in the various tissues and transformed the gene into the model plant, *A. thaliana* (Colombia ecotype-0). The gene *CotAD_24498* was analyzed in various tissues of the upland cotton, *G. hirsutum*. This was carried out in order to determine its relative abundance within the plant. We found that the gene was more abundantly expressed in the reproductive tissues, more specifically in the petal and stamen (Figure 7A). In addition, we further carried out treatment on cotton seedlings after three true leaves stage under drought stress (PEG6000_15%) the samples for RNA extraction and qRT-PCR analysis were obtained from leaf, root and leaves at intervals of 0 h, 3 hr, 6 hr, 12 hr and 24 hr of post stress treatment. In all the three tissues, 6 hr marked the peak up-regulation of the gene, and then a gradual decline was observed with increase in time of stress exposure. The gene exhibited a significant up regulation in the root as compare to leaf and stem tissues (Figure 7B). We successfully transformed 9 lines with overexpressed gene *CotAD_24498* (Figure 7C), out the nine (9) lines, three (3) lines showed the highest level of overexpression and were further used in the investigation of the potential of the gene in the transgenic lines under drought stress conditions (Figure 7D).

### Overexpression of CotAD_24498 in plants promote root growth and confers tolerance to drought stress tolerance

**Figure 8:**
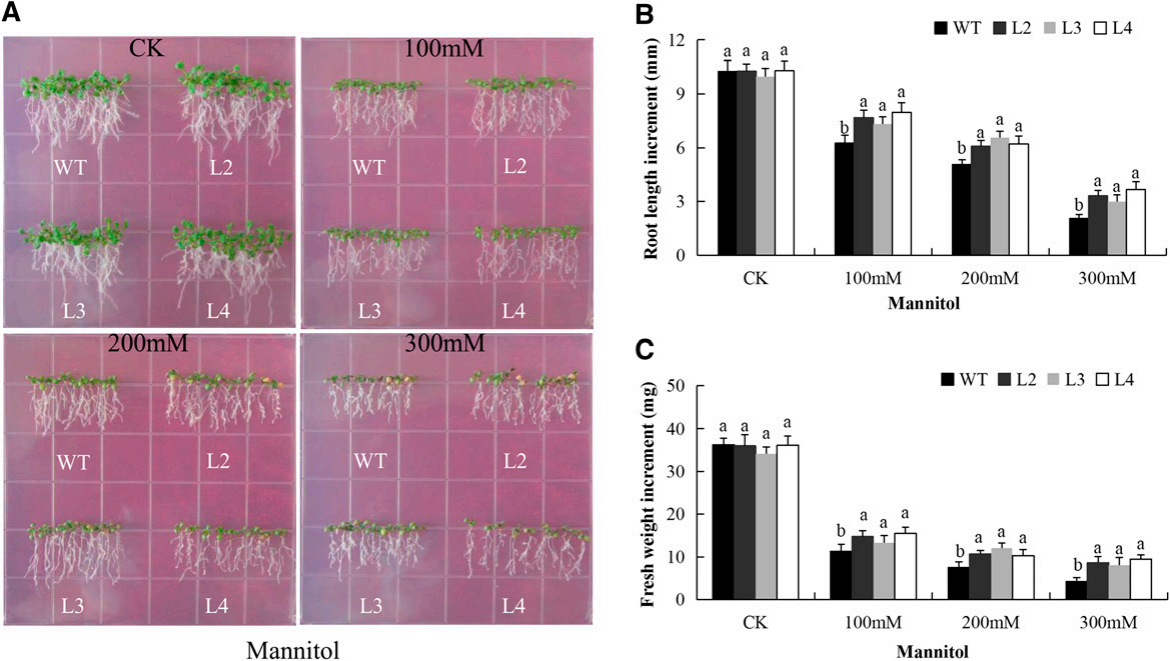
Overexpression of *CotAD_24498* enhances root growth and drought stress tolerance in *Arabidopsis* transgenic lines (A) *CotAD_24498* overexpressing and WT plants were grown vertically in 0.5 Murashige and Skoog (MS) medium supplemented with 0, 100, 200 and 300 mM mannitol and incubated for 6 days. (B). Root elongation comparisons on 0.5 MS put at normal and osmotic stress for 6 days. The seedlings were scored and photographed after 6 days post germination. (C). Quantitative determination of fresh weight biomass of wild-type (WT) and both transgenic lines (L2, L3 and L3) after 6 days post germination at normal and drought stress condition. In (B, C,), each experiment was repeated three times. Bar indicates standard error (SE). Different letters indicate significant differences between wild-type and transgenic lines (ANOVA; *P* < 0.05). CK: normal conditions.

Increased primary root growth and overall plant fresh biomass are indicators of tolerance to various abiotic stresses in which plants are exposed to (Verslues *et al.* 2006; Jisha *et al.* 2013). We sought to investigate the response of the transgenic lines and the wilt type to drought stress condition in relation to primary root length elongation and fresh biomass accumulation. The transgenic lines showed enhanced performance with relatively increased primary root growth and with higher fresh biomass increment compared to the wild type under drought stress condition. The drought stress was imposed by exposing the transgenic lines to different concentrations of mannitol 0 mM, 100 mM, 200 mM and 300 mM for a period of six (6) days. Under osmotic stress, highest level of root length assays and fresh biomass accumulations was observed at 100 mM of mannitol concentration (Figure 8B). The transgenic lines had significantly higher primary root length and fresh biomass accumulation (Figure 8C), an indication that the photosynthetic processes were not impaired by the drought stress as compared to the wilt type.

### Transcripts Investigation of Drought Stress-Responsive Genes

**Figure 9:**
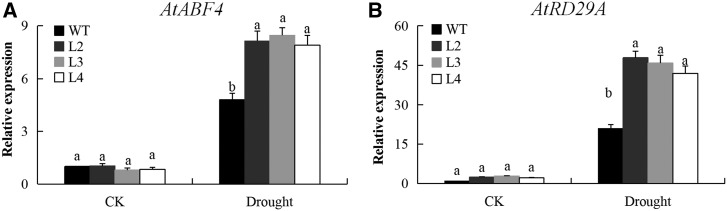
Expression levels of drought stress-responsive genes (*ABF4* and *RD29A*) in transgenic lines and wild-type. *Arabidopsis ACTIN2* was used as the reference gene mean values with *±* SD. * *P* < 0.05 as calculated by Student’s *t*-test.

The root appears to be the most relevant organ for breeding drought stress tolerance (Henry 2013). Underlying the ABA-mediated stress responses is the transcriptional regulation of stress-responsive gene expression (Giraudat *et al.* 1994). Numerous genes have been reported that are up-regulated under stress conditions in vegetative tissues, these include a class of genes known as *LEA* genes, which are expressed abundantly in developing seed under normal conditions, osmolyte biosynthetic genes, and genes of general cellular metabolism. We undertook to check the expression of two known abiotic stress responsive genes on the transgenic lines (L2, L3 and L4) and the wild types when the plants are exposed to drought condition. The result showed that the stress responsive genes were highly up-regulated in the transgenic lines as opposed to the wild type (Figure 9). The result obtained was in agreement to the result obtained when the various *LEA2* genes were analyzed through qRT-PCR on the tissues obtained from two upland cotton genotypes. More genes were found to be up regulated on the various tissues of the more tolerant genotype as opposed to the less tolerant. Constitutive expression of *RD29A* and *ABF4* demonstrated enhanced drought tolerance in the transgenic *Arabidopsis* plants.

### Oxidants and antioxidant determination in the transgenic lines

**Figure 10:**
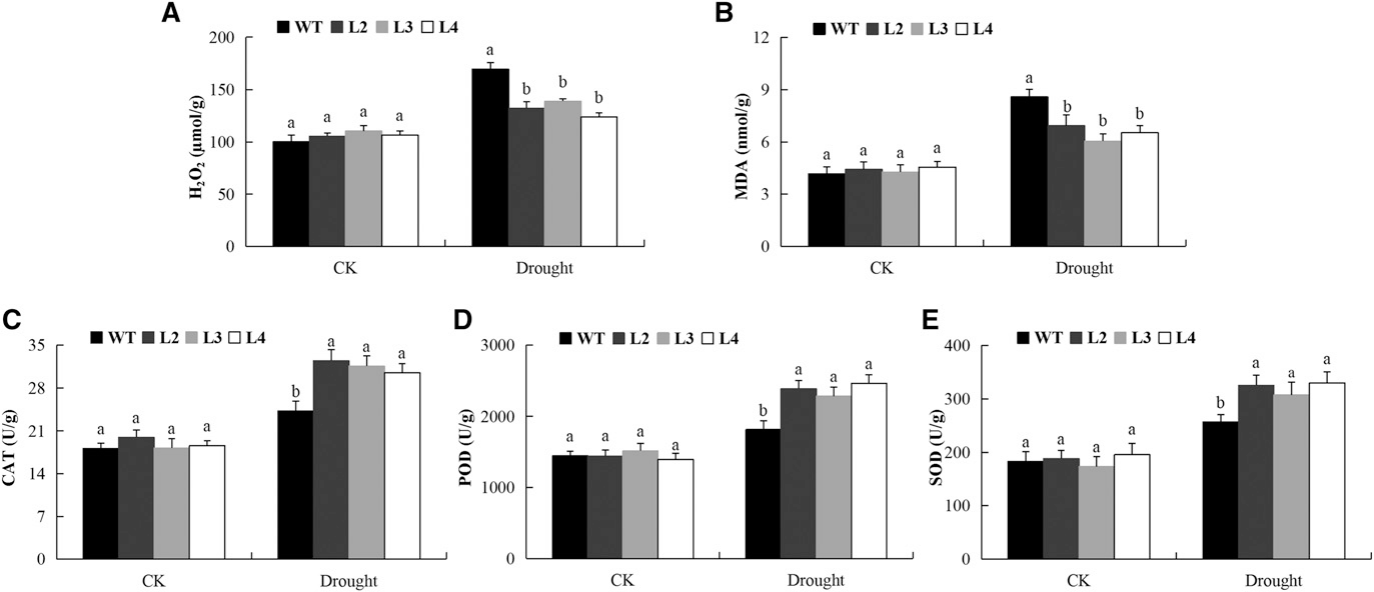
determination of the oxidants and antioxidants in the transgenic lines under stress condition (A) Determination of hydrogen peroxide (H_2_O_2_) accumulation in leaves of wild-type (WT) and both transgenic lines (L2, L3, and L4) after 8-days drought stress (B) Determination of MDA accumulation in leaves of wild-type (WT) and both transgenic lines (L2, L3, and L4) after 8-days drought stress; (C) Catalase (CAT) activity, (D) peroxidase (POD) activity and (E) superoxide dismutase (SOD) activity. Data are means ± SE calculated from three replicates. Different letters indicate a significant difference between the WT and both transgenic lines (ANOVA; *P* < 0.05).

In order to understand the role of the transformed *LEA2* genes in the transgenic lines in relation to drought stress. We carried out the analysis of the various oxidants and antioxidants measurements in the leaves of the transgenic lines and the wild type. The levels of oxidants were significantly reduced in the transgenic lines compared to the wild type (Figure 10A-B). When plants are exposed to drought the level of ROS increases, which results into oxidative stress. MDA concentration provides a measure on the damage caused on the membrane lipids due to oxidative stress (Jain *et al.* 2001). The significant reduction in MDA and H2O2 in the leaf tissues of the transgenic lines showed that the transformed gene had a regulatory role in controlling various biological pathways geared toward detoxification of the reactive oxygen species in the cells. In addition, we quantified the levels of various antioxidants, SOD, POD and CAT. In all the three antioxidants, there was significant increased levels in the transgenic lines (L1, L2 and L3) compared to the wild type (Figure 10 C-D). The increased levels of the antioxidants showed that the transgenic lines had a higher ability to tolerant drought stress compared to the wild types. The results obtained in this research, correlates to previous findings, in which drought stressed wheat plants were found to have higher accumulation of oxidants levels (Luna *et al.* 2005). More tolerant plants genotypes have ability to induct more of the antioxidants such as the CAT, POD and SOD in order to scavenge on the excess ROS and other deleterious molecules released by the cells due to stress condition (Bian and Jiang 2009).

### Conclusions

In this study, the identification, phylogenetic relationships, miRNA targets, cis promoter analysis, GO functional annotation and exon/intron structures of *LEA2* genes family members were evaluated in upland cotton, *Gossypium hirsutum*, and the tissue expression pattern of the two tetraploid cotton species, *G. hirsutum* (drought sensitive) and *G. tomentosum* (drought tolerant) were detected under drought stress. The abundance of *LEA2* genes and unique gene structure reported in this work provide a solid foundation for future research to understand the evolution of *LEA2* gene family and the potential functional role of the 157 *LEA2* genes in plants under drought stress condition. Since the discovery of *LEA* genes, little work has been reported on *LEA* genes as a whole in upland cotton. The transformation and expression analysis of the transformed *LEA2* gene indicated that the *LEA2* genes have a profound role in enhancing drought stress tolerance. The transgenic lines L2, L3 and L4 exhibited superior performance compared to the wild type. The roots were significantly longer than the wild type under drought stress condition; similarly, the levels of oxidants in the levels were significantly reduced while the antioxidants levels were higher in the leaves of the transgenic lines compared to the wild type. An indication that the transgenic plants had a higher capacity to regulate the oxidative stress as opposed to the wild type (WT). The genes could be promoting growth of the root cells under limited water condition. Primary root growth is linked to drought stress tolerance; due to increased surface area of the roots thus improving its ability maximally absorb any little moisture available. Deep or extensive root growth is a trait known for most of the xerophytic plants (Brunner *et al.* 2015).
